# Effect of transcutaneous electrical nerve stimulation on patients after coronary artery bypass grafting: a systematic review and meta-analysis

**DOI:** 10.3389/fcvm.2026.1690565

**Published:** 2026-02-05

**Authors:** Enyu Zhang, Jihe Kang, Yan Liu, Lulu Wang, Bo Wan, Xiaoling Li

**Affiliations:** Department of Rehabilitation Medicine, The Second Hospital of Lanzhou University, Lanzhou, Gansu, China

**Keywords:** analgesics, coronary artery surgery, lung function, postoperative pain, transcutaneous electrical nerve stimulation

## Abstract

**Objective:**

This study aims to systematically evaluate the potential effects of transcutaneous electrical nerve stimulation (TENS) on analgesic consumption, pain relief, and pulmonary function outcomes in patients following coronary artery bypass grafting (CABG).

**Methods:**

A systematic literature search was conducted across four core databases—PubMed, Embase, Web of Science, and the Cochrane Library. Methodological quality was evaluated using the Cochrane Risk of Bias (RoB 2.0) tool, and the certainty of evidence was evaluated using the GRADE system. For statistical analysis, standardized mean differences (SMDs) with 95% confidence intervals (CIs) were calculated for continuous variables. Heterogeneity was assessed via the *I*^2^ statistic (threshold: 50%), with fixed-effects models applied when *I*^2^ ≤ 50% or random-effects models applied when *I*^2^ > 50%. Subgroup analyses stratified by intervention duration were performed for outcomes demonstrating significant heterogeneity (*I²* ≥ 50%).

**Results:**

First, regarding pain management, TENS demonstrated greater efficacy in postoperative acute pain at rest (within 12 h) than control interventions (SMD = −1.02, 95% CI: −0.23–2.28; *P* = 0.11), albeit with high heterogeneity (*I*^2^ = 88.50%). Postoperative chronic pain at rest (5 days) was more pronounced (SMD = −2.00, 95% CI: −4.15–0.15; *P* = 0.07). Second, in terms of pulmonary recovery, TENS significantly improved forced expiratory volume within 1 s (FEV_1_: SMD = 0.85, 95% CI: 0.43–1.26; *P* = 0.00) but not forced vital capacity (FVC: SMD = −1.02, 95% CI: −0.23–2.28; *P* = 0.11), with heterogeneity levels of 54.00% and 88.50%, respectively. Third, regarding analgesic use, TENS reduced postoperative opioid consumption (SMD = −4.23, 95% CI: −7.31 to −1.15; *P* = 0.007), although heterogeneity remained high (*I*^2^ = 96.20%).

**Conclusion:**

The current evidence preliminarily suggests that TENS may reduce postoperative analgesic dependence and modestly improve pulmonary function in patients following CABG; however, it has not demonstrated a statistically significant advantage in pain relief. Given the substantial heterogeneity across studies, these findings should be interpreted with caution. Future large-scale RCTs with standardized protocols are needed to validate these findings.

**Systematic Review Registration:**

https://www.crd.york.ac.uk/PROSPERO/view/CRD42024594786, PROSPERO CRD42024594786.

## 1 Introduction

Coronary artery bypass grafting (CABG) is a critical surgical intervention for patients with severe coronary artery disease (1). However, postoperative complications, such as immunosuppression, heightened inflammatory responses, decreased exercise tolerance, and opioid dependence, can significantly hinder patient recovery (2). Studies indicate that patients undergoing CABG with cardiopulmonary bypass exhibit markedly elevated serum levels of proinflammatory factors such as IL-6 and CRP, thereby increasing the risk of postoperative infection. Concurrently, excessive sympathetic excitation triggers peripheral vasoconstriction, further restricting muscle blood supply and oxygen delivery, which contributes to reduced postoperative exercise capacity. Traditional analgesic strategies relying on high-dose opioids may induce adverse events, including respiratory depression and gastrointestinal dysfunction, with persistently high incidence rates (3). Thus, exploring safe, non-pharmacological interventions capable of synergistically addressing postoperative multisystem dysfunction has become an urgent clinical priority.

Transcutaneous electrical nerve stimulation (TENS) is a non-invasive physical therapy that exerts multitarget effects by modulating the neuro-immuno-endocrine network. Its proposed mechanisms include (1) activation of large-diameter nerve fibers to inhibit pain signal transmission (gate control theory); (2) stimulation of endogenous opioid peptide release, alleviating inflammatory responses and central sensitization; and (3) regulation of autonomic balance to suppress sympathetic hyperactivity, thereby improving peripheral blood perfusion (4). Recent clinical studies have validated the potential role of TENS in post-CABG management. Specifically, TENS has been shown to reduce postoperative IL-6 and CRP levels while decreasing the incidence of pulmonary infection. In addition, sympathetic ganglion stimulation has been associated with enhanced femoral artery blood flow and extended walking distance (5). Furthermore, a combined distal‒proximal acupoint stimulation strategy has been reported to reduce intraoperative sufentanil requirements and shorten the duration of mechanical ventilation. These findings suggest that TENS may ameliorate CABG-related pathophysiological disturbances through multiple pathways.

However, the existing evidence has several notable limitations. First, high heterogeneity in stimulation parameters (e.g., frequency, intensity, and acupoint selection) without standardization has led to inconsistent conclusions regarding efficacy. For example, sham stimulation controls show no significant hemodynamic improvements, whereas specific acupoint combinations demonstrate synergistic effects. Second, most studies are limited by small sample sizes, and risk-of-bias assessments have revealed inadequate allocation concealment and blinding in some RCTs (6). In addition, systematic reviews predominantly focus on single outcomes (e.g., immune function or pain relief) and lack a comprehensive evaluation of the holistic benefits of TENS.

Therefore, this study aims to comprehensively integrate the best available evidence through a systematic review and meta-analysis to evaluate the effects of TENS on multidimensional clinical outcomes in patients undergoing CABG. This study not only focuses on its analgesic and opioid-sparing effects but also examines the impact of TENS on lung function. The results of this study may provide evidence-based support for the standardized, precision-guided implementation of TENS in ERAS protocols following CABG, thereby contributing to improved postoperative recovery and reduced healthcare costs and offering potential clinical relevance for future practice.

## 2 Materials and methods

### 2.1 System evaluation registration and execution standards

This study was preregistered with the International Prospective Register of Systematic Reviews (PROSPERO; Registration No. CRD42024594786) and was conducted in strict accordance with the methodological standards of the Preferred Reporting Items for Systematic Reviews and Meta-Analyses (PRISMA) 2020 Statement (7).

### 2.2 Literature retrieval strategy

This study primarily searched four English databases (PubMed, Embase, Web of Science, and the Cochrane Library) and clinical trial registration platforms (ClinicalTrials.gov), with parallel retrieval of gray literature (including conference abstracts, dissertations, and ongoing projects), with the search covering all records published up to December 2025. The search keywords included “coronary artery bypass grafting” and “transcutaneous electrical nerve stimulation.” Boolean operators (AND/OR) were used to combine controlled vocabulary (MeSH/Emtree terms) with free-text keywords, whereas truncation symbols (*) were applied to capture lexical variations. The specific search strategies for each database are detailed in Supplementary Table S1.

### 2.3 Inclusion and exclusion criteria

#### 2.3.1 Inclusion criteria

Population (P): Adult patients aged 18–80 years who underwent CABG for myocardial revascularization.
Intervention (I): Postoperative application of high-frequency TENS with parasternal electrode placement (positioned approximately 5 cm from the sternal midline). The parameters included stimulation intensity adjusted to individual tolerance, a high-frequency modulation range of 50–150 Hz, a pulse width of 50–250 μs, and a single-session duration of 20–60 min.
Control (C): Parallel control groups comprising (1) an active control group receiving guideline-based standard postoperative analgesia (e.g., opioids combined with NSAIDs) and (2) a placebo group using non-current-output TENS devices with procedures identical to those of the intervention group.
Outcomes (O): Primary outcomes included postoperative pain intensity at rest, quantified using a visual analog scale (VAS, 0–10 cm), and postoperative opioid consumption (cumulative doses converted to morphine milligram equivalents). Secondary outcomes included measures of lung function (FEV_1_ or FVC after surgery), assessed using a standardized lung-function device.
Study design (S): Only randomized controlled trials were included.

#### 2.3.2 Exclusion criteria

Studies involving non-human subjects (e.g., animal experiments or *in vitro* studies).
Unpublished data, duplicate publications, or studies with inaccessible full texts.
Studies not adhering to the PICOS framework, including non-randomized trials, case reports, commentaries, and meta-analyses (non-original research).

### 2.4 Literature screening process

This study implemented a dual-independent screening process for literature selection. Two researchers (ZEY and KJH) systematically removed duplicate records from the search results using EndNote X9 software. Automated deduplication was initially performed on the basis of predefined field-matching criteria (title, author, publication year, etc.), followed by manual verification of suspected duplicates. Subsequently, title/abstract screening was performed in parallel according to the prespecified inclusion criteria. Articles meeting the initial inclusion criteria were assessed in full text by two reviewers. Disputes arising during this process were ultimately resolved by third-party arbitration.

### 2.5 Data extraction

The research team utilized a standardized data collection template to systematically extract information from the included literature. Two senior researchers (ZEY and KJH) independently executed double-masked data extraction to ensure accuracy (8). To enhance interstudy comparability, the team prioritized the collection of baseline characteristics of the study populations, including age, sex distribution, body mass index (BMI), and intervention protocols with implementation standards for both the experimental and control groups. The efficacy evaluation system incorporated three core metrics: (1) pain relief assessed using the VAS; (2) pulmonary function improvement quantified by forced expiratory volume in 1 s (FEV_1_) and forced vital capacity (FVC); and (3) opioid analgesic consumption. For studies with missing data, the team contacted the corresponding authors of the original studies via email to obtain unpublished information (8). Disputes arising during this process were ultimately resolved by third-party arbitration.

### 2.6 Literature quality evaluation

Two researchers, ZEY and KJH, independently assessed the risk of bias and graded the quality of evidence. Any disputes arising during this process were resolved by third-party arbitration.

#### 2.6.1 Bias risk assessment

This study assessed the quality of the included RCTs using the Cochrane Risk of Bias in Randomized Controlled Trials tool, version 2.0 (RoB2.0) (9). Five core domains were systematically evaluated: (1) the scientific rigor of randomization procedures; (2) the standardization of intervention implementation; (3) the completeness of outcome data; (4) the accuracy of measurement methods; and (5) the selective reporting of results. Each domain was evaluated using three-tier rating criteria (low risk, some concern, or high risk) according to standardized guidelines. Assessors were required to document the rationale for each judgment for each dimension. Comprehensive risk-of-bias conclusions were derived by synthesizing evaluations across all five domains.

#### 2.6.2 GRADE evidence quality

The Grading of Recommendations Assessment, Development, and Evaluation (GRADE) method was used to assess the quality of evidence for each outcome. Evidence quality could be degraded by specific limitations of the original studies, including risk of bias, inconsistency, indirectness, imprecision, and publication bias. According to the GRADE guidelines, the overall quality of evidence was categorized into four levels: high, moderate, low, and very low (10). Two independent reviewers used the GRADEpro GDT software to assess the quality of evidence for each outcome (e.g., postoperative resting pain intensity measured by the VAS, cumulative opioid consumption, and pulmonary function parameters such as FEV₁ and FVC). The final certainty ratings of evidence—categorized as high, moderate, low, or very low—are clearly presented in a summary-of-findings table in the Results section.

### 2.7 Statistical analysis

Data synthesis and statistical analyses were performed using StataSE 15.0 software (StataCorp LP, Texas, USA). Interstudy heterogeneity was assessed using Higgins' *I*^2^ statistic and Cochran's *Q* test (11, 12). When significant heterogeneity was detected (*I*^2^ values > 50% and *P*_H_ < 0.05), a random-effects model was applied; otherwise, a fixed-effects model was used. Forest plots were used to present the pooled results and their corresponding 95% CIs. The primary outcome measures included resting pain intensity (quantified using the VAS) and pulmonary function parameters [measured by forced expiratory volume in 1 s (FEV_1_) and forced vital capacity (FVC)]. Sensitivity analyses were performed by stepwise exclusion of individual studies to assess the robustness of the findings. Due to the limited number of included studies, the following analysis methods could only be attempted as far as possible: first, subgroup analyses and meta-regression analyses based on the type of intervention in the experimental group were conducted to explore the heterogeneity. Second, publication bias among the included studies was quantitatively assessed using funnel plots in combination with Egger's test.

## 3 Results

### 3.1 Literature search results

**Figure 1:**
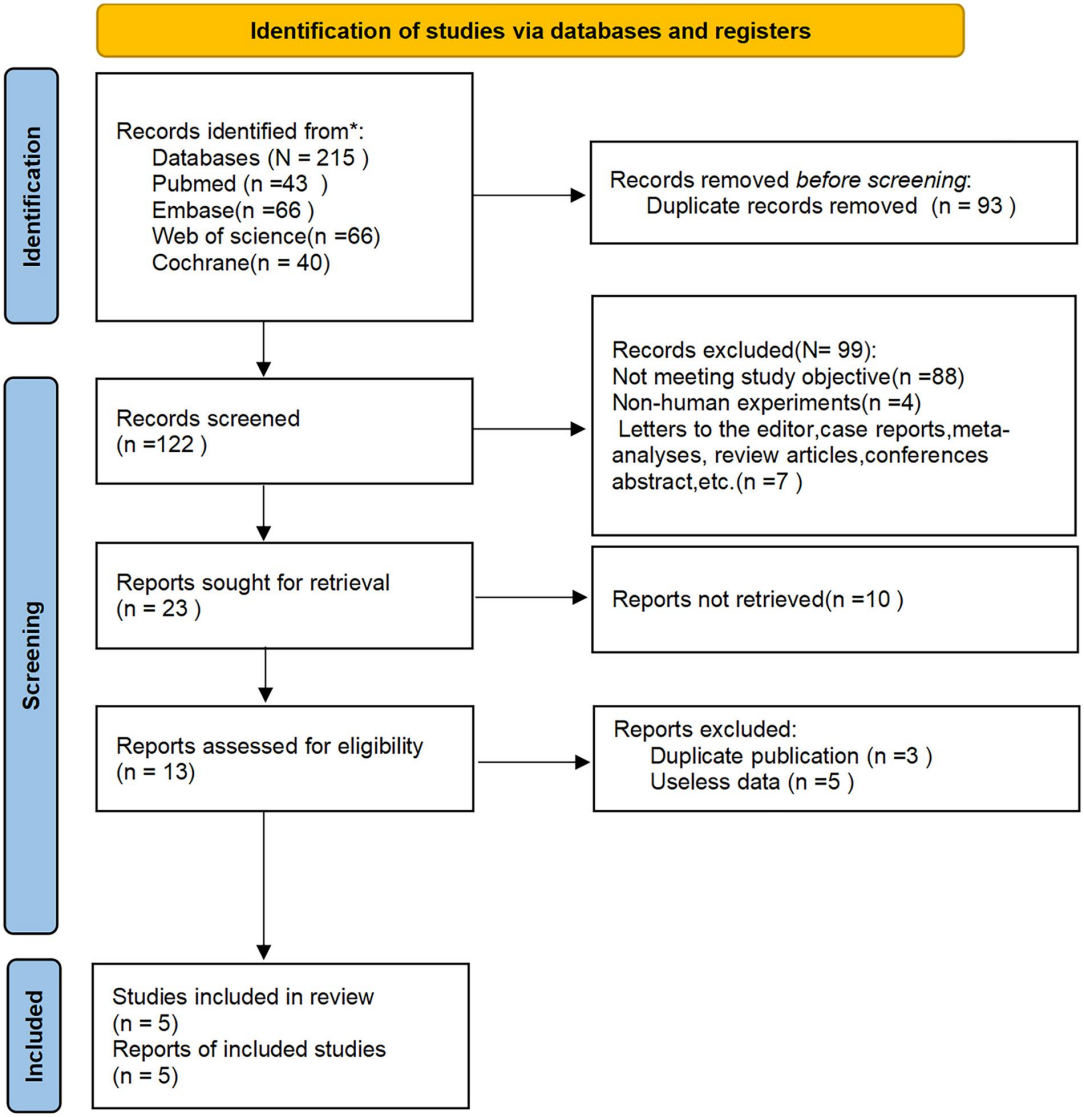
PRISMA flow diagram of the study selection process.

Through a systematic search process, 215 potentially relevant articles were initially identified. After removal of duplicate studies using reference management software (*n* = 93), 122 records remained for title/abstract screening. Application of the predefined exclusion criteria through dual independent screening resulted in the exclusion of 99 publications, leaving 23 articles for full-text evaluation. Following critical appraisal of the full texts, 18 studies were excluded due to methodological limitations, leaving five RCTs (13–17) that met all prespecified inclusion criteria. The screening process strictly adhered to the PRISMA 2020 guidelines, as illustrated in Figure 1.

### 3.2 Basic characteristics of the included studies

Five RCTs (13–17) were included in this study, comprising a total of 327 patients, primarily middle-aged and elderly individuals. The mean age across study populations ranged from 58.4 to 67.8 years. The frequency modulation of TENS was 100 Hz, and the pulse duration was set between 100 and 250 μs across different clinical studies. Two studies used TENS combined with conventional analgesia in the experimental group (15, 16), two studies employed TENS combined with patient-controlled analgesia (PCA) as the experimental group (14, 17), and only one study investigated TENS as a standalone experimental intervention (13). The basic characteristics of the included studies are detailed in Table 1.

**Table 1:** Basic characteristics of the included studies.

Author	Publication year	Study design	Country	Experimental group (TENS)			Control group		
				Sample size	Age (years)	Intervention	Sample size	Age (years)	Intervention
Cipriano et al. (13)	2014	RCT	USA	20	62 ± 4	TENS: The frequency and pulse width were preset to 100 Hz and 150 μs, respectively; the electrodes were attached to the C7 and T4 vertebral bodies, about 3 cm from the midline; the treatment was given in four 30-min sessions per day for 4 consecutive days	18	66 ± 3	Sham electrical stimulation: Pulse mode without the analgesic effect, and other operations were the same as those in the experimental group
Dontaille et al. (14)	1997	RCT	UK	31	59.7 ± 7.5	TENS combined with patient-controlled analgesia (PCA)	28	59.1 ± 9.0	Placebo TENS combined with PCA
						TENS: The frequency and pulse width were set at 100 Hz and 100 μs, respectively; the electrode patch was positioned at the midpoint of the vertical length of the sternal incision, next to the wound dressing, and approximately an inch (2.54 cm) from the center of the incision; treatment lasted for 1 h			Placebo TENS: There was no current output, and the other operations were the same as in the experimental group
						PCA: Morphine			PCA: Morphine
Jahangirifard et al. (15)	2018	RCT	Iran	50	58.4 ± 8.1	TENS combined with conventional analgesia	50	60.1 ± 6.6	Conventional analgesic therapy: Continuous intravenous infusion of morphine (1 mg/ h); a 100 mg diclofenac suppository was used
						TENS: The frequency was set at 100 Hz, and the pulse width was set at 250 μs; the electrodes were attached to the proximal and distal ends of the sternal incision, approximately 2.5 cm (1 in.) away from the incision and did not interfere with the wound dressing; the treatment was given in four 30-min sessions per day for 3 consecutive days			
						Conventional analgesic therapy: Continuous intravenous infusion of morphine (1 mg/h); a 100 mg diclofenac suppository was used			
Luchesa et al. (16)	2009	RCT	Brazil	15	/	TENS combined with conventional analgesia	15	/	Placebo TENS combined with conventional analgesic treatment
						TENS: The frequency and pulse width were preset to 100 Hz and 125 μs, respectively; the first pair of electrodes was placed 3 cm below the sternoclavicular joint, 3 cm from the sternal incision; the second pair of electrodes was placed 2 cm above the xiphoid process and 3 cm from the sternal incision; the treatment was given twice daily for 50 min for 5 consecutive days			Placebo TENS: There was no current output, and the other operations were the same as in the experimental group
						Conventional analgesic treatment: Conventional drugs			Conventional analgesic treatment: Conventional medications
Solak et al. (17)	2009	RCT	Turkey	25	64.5 ± 6.93	Continuous TENS combined with PCA	25	65.0 ± 6.4	Placebo TENS combined with PCA
						Continuous TENS: The frequency and pulse width were set at 100 Hz and 100 μs, respectively; two electrodes (channel 1) were placed on one side of the incision, and the other two electrodes (channel 2) were placed on the other side of the incision; the electrode edge was about 1 cm away from the suture line; treatment was continued for 24 h			Placebo TENS: There was no current output, and the other operations were the same as in the experimental group
						PCA: Morphine sulfate standard solution (1 mg/mL)			PCA: Morphine sulfate standard solution (1 mg/mL)
				25	67.8 ± 8.60	Intermittent TENS combined with PCA	25	65.0 ± 6.4	Placebo TENS combined with PCA
						Continuous TENS: The frequency and pulse width were set at 100 Hz and 100 μs, respectively; two electrodes (channel 1) were placed on one side of the incision, and the other two electrodes (channel 2) were placed on the other side of the incision; the electrode edge was about 1 cm away from the suture line; stimulation for 1 h → rest for 1 h → restimulation for 1 h (i.e., 1:1 stimulus–rest cycle), with a total duration of 24 h			Placebo TENS: There was no current output, and the other operations were the same as in the experimental group
						PCA: Morphine sulfate standard solution (1 mg/mL)			PCA: Morphine sulfate standard solution (1 mg/mL)

### 3.3 Literature quality evaluation

#### 3.3.1 Bias risk assessment

A systematic evaluation based on the RoB2.0 assessment framework showed variability in methodological quality across the five included RCTs. Jahangirifard et al. (15) used computer-generated random sequences, while allocation concealment was described using sealed envelopes only in the study of Dontaille et al. (14). In general, the overall risk of bias was low for Jahangirifard et al. (15) and Dontaille et al. (14). In contrast, Luchesa et al. (16) and Solak et al. (17) demonstrated potential methodological limitations in the randomization process and were therefore rated as having “some concerns.” Cipriano et al. (13) were classified as having a high risk due to obvious deficiencies in the implementation of the intervention. Detailed evaluation results are presented in Supplementary Figures S1, S2.

#### 3.3.2 GRADE assessment

According to the GRADE evaluation, the overall quality of evidence for all outcomes included in this study was rated as moderate. The primary reason for downgrading of all outcomes was “serious imprecision,” reflected by wide confidence intervals and the inability to completely rule out the possibility of no effect. However, the consistent direction of the effect estimates supports the potential benefits of TENS. No serious concerns were identified regarding the risk of bias, inconsistency, or indirectness. All outcomes were considered “important” or “critically important” clinical questions. Although existing evidence suggests that TENS has a positive effect on postoperative pain relief, pulmonary function improvement, and analgesic use reduction, the results are uncertain due to the limited sample size. Future high-quality, large-sample RCTs are still necessary to further strengthen the level of evidence and verify the findings of this study. Detailed results are presented in Supplementary Table S2.

### 3.4 Meta-analysis results

#### 3.4.1 Postoperative acute pain at rest (within 12 h)

The studies by Dontaille et al. (14) and Solak et al. (17) evaluated changes in pain over a 12-h period following TENS treatment. Both studies employed similar intervention protocols, comparing TENS combined with PCA vs. placebo TENS combined with PCA. Among them, Solak et al. (17) reported two different pulse modulation modes of TENS (continuous and intermittent). Therefore, a total of three datasets were included in the pooled results of this study.

**Figure 2:**
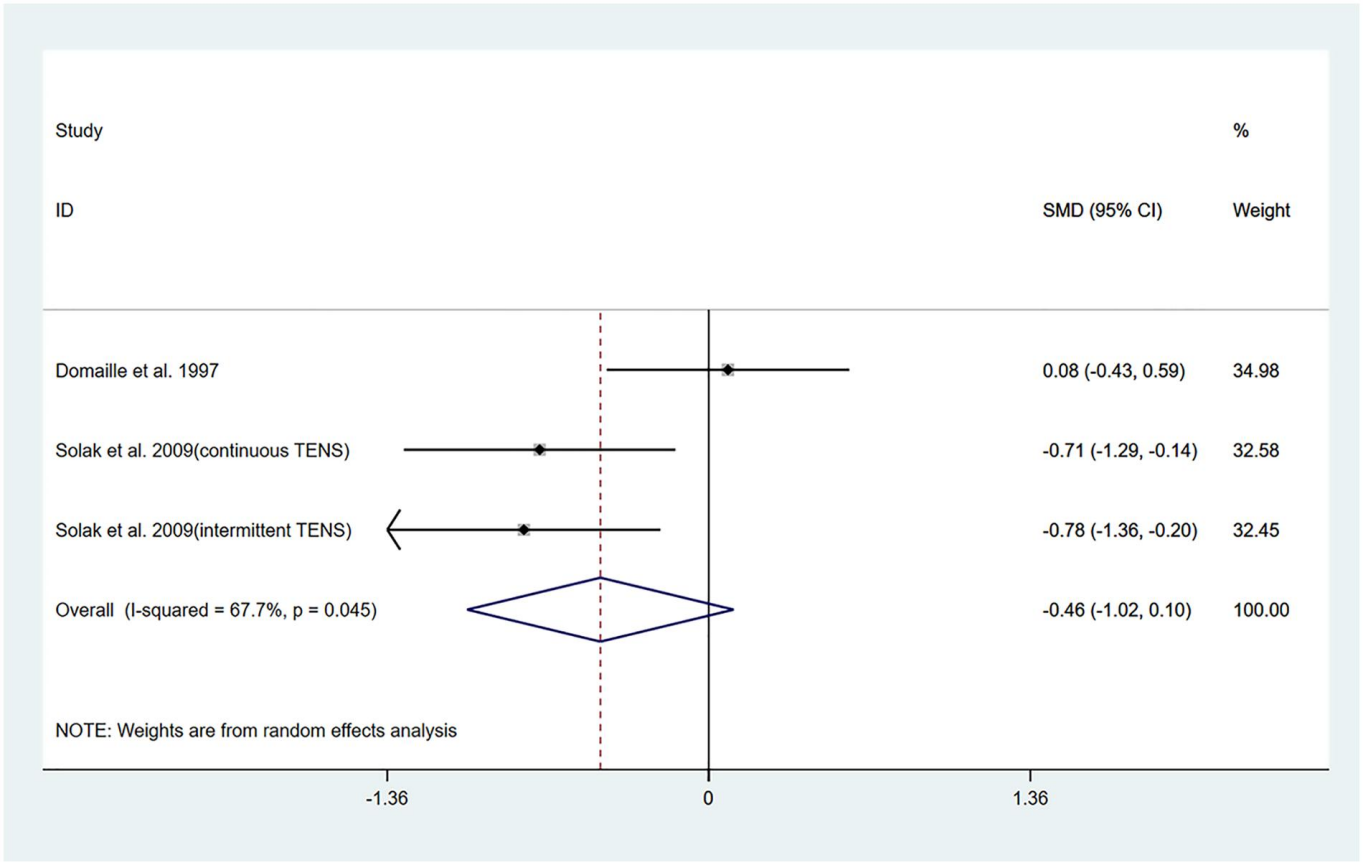
Resulting forest plot of postoperative acute pain at rest (within 12 h).

The results of the heterogeneity test showed that *I^2^* = 67.70% and P_H_ = 0.045. Therefore, a random-effects model was applied for pooling, and the pooled analysis yielded the following: standardized mean difference (SMD) = −0.46, 95% CI (−1.02, 0.10), *Z* = 1.59, and *P* = 0.11. These results indicated that although TENS reduced VAS scores within 12 h posttreatment compared to the control group, this reduction was not statistically significant (Figure 2). Sensitivity analyses demonstrated the robustness of the findings of the study (Supplementary Figure S3). Owing to the limited number of studies included, publication bias results could not be assessed.

#### 3.4.2 Postoperative chronic (5 days) pain at rest

The studies by Cipriano et al. (13) and Luchesa et al. (16) evaluated changes in pain after 5 days of TENS treatment. Two datasets were included in the pooled analysis.

**Figure 3:**
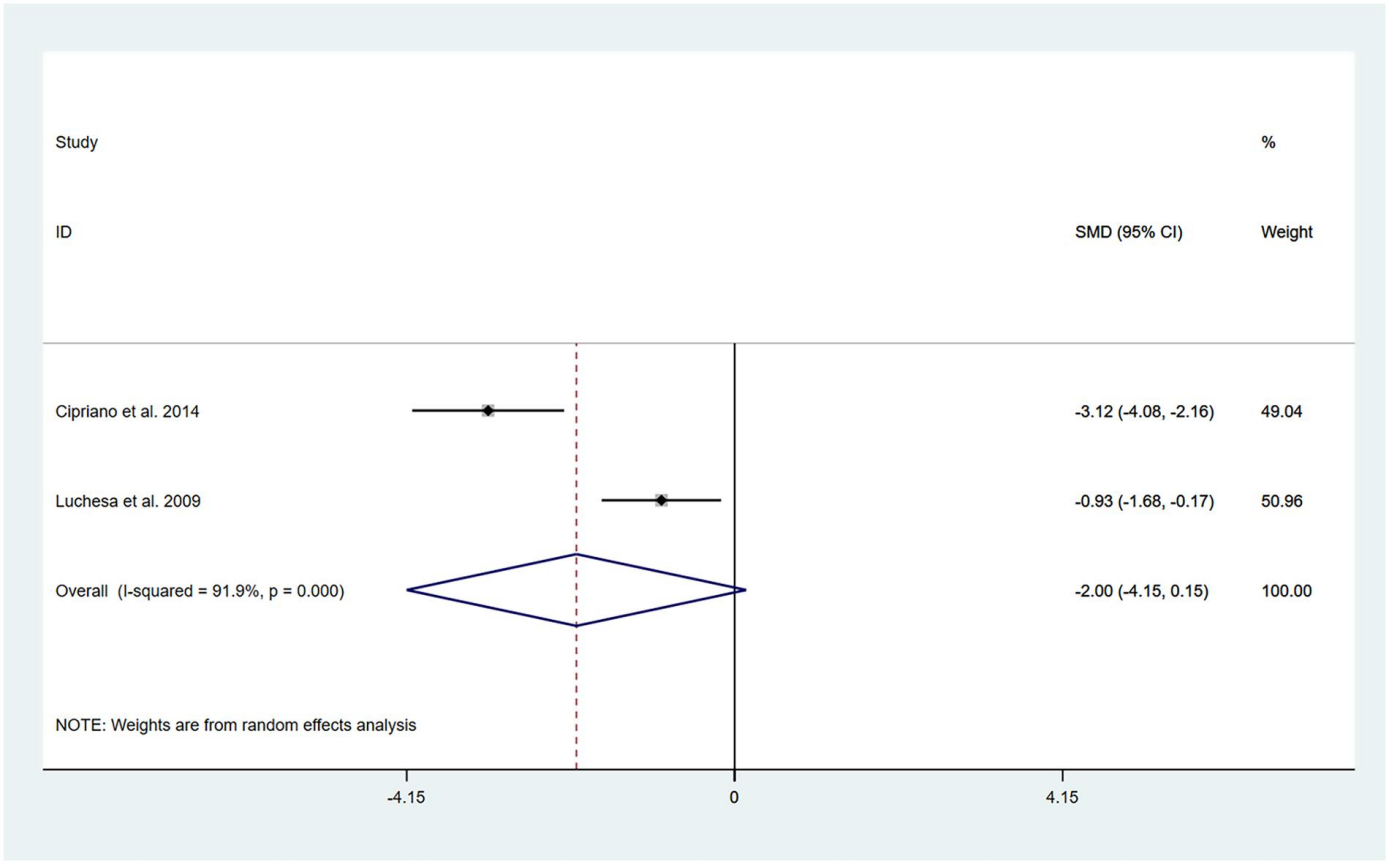
Resulting forest plot of postoperative chronic pain at rest (5 days).

The heterogeneity test results showed that *I*^2^ = 91.90% and P_H_ = 0.00. Therefore, a random-effects model was applied for the pooling, and the pooled analysis yielded the following: SMD = −2.00, 95% CI (−4.15, 0.146), *Z* = 1.83, and *P* = 0.07. The results indicated that, compared with the control group, TENS was capable of lowering the VAS score at 5 days posttreatment; however, the difference did not reach statistical significance (Figure 3). Sensitivity analyses revealed that the findings of this study are robust (Supplementary Figure S4). Owing to the limited number of included studies, publication bias results could not be assessed.

In this analysis, we observed a high level of heterogeneity, and there were differences in intervention protocols between the two included studies. Detailed information is provided in Table 1. Therefore, the differences in the intervention measures might be one of the main reasons for the high heterogeneity.

#### 3.4.3 Postoperative pain medication consumption

The studies conducted by Cipriano et al. (13) and Solak et al. (17) evaluated the anesthetic drug dosage consumed by patients after receiving TENS treatment.

Among them, Solak et al. (17) reported two different pulse modulation methods of TENS: (continuous and intermittent). Detailed information is provided in Table 1. A total of three datasets were included in the pooled results of this study.

**Figure 4:**
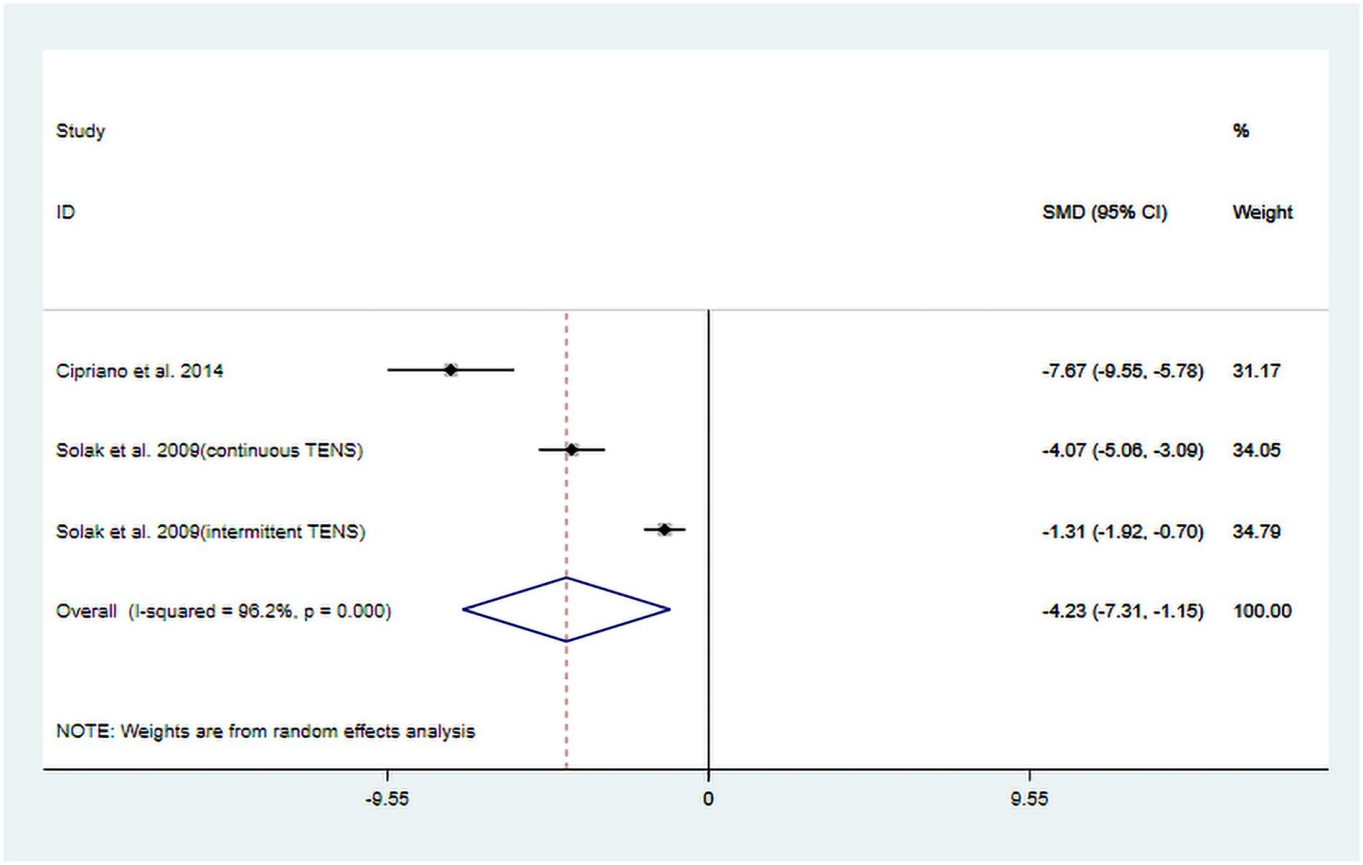
Resulting forest plot of postoperative pain medication consumption.

The heterogeneity test results showed that *I^2^* = 96.20% and P_H_ = 0.00. Therefore, a random-effects model was applied for pooling, and the pooled analysis yielded the following: SMD = −4.23, 95% CI (−7.31, −1.15), *Z* = 2.69, and *P* = 0.007. The results indicate that, compared to the control group, TENS significantly reduced postoperative anesthetic drug requirements (Figure 4). Sensitivity analyses demonstrated that the study findings are robust (Supplementary Figure S5). However, due to the limited number of studies included, publication bias results could not be assessed. A high degree of heterogeneity in outcomes was noted, and the intervention strategies varied slightly across the two studies. Cipriano et al. (13) applied TENS to the treatment group, whereas Solak et al. (17) utilized TENS in conjunction with PCA. These variations in intervention protocols are likely a primary contributor to the observed heterogeneity.

#### 3.4.4 Postoperative pulmonary function

##### 3.4.4.1 FEV_1_

The studies by Jahangirifard et al. (15), Luchesa et al. (16), and Solak et al. (17) evaluated FEV_1_ after TENS treatment. In the studies by Jahangirifard et al. (15) and Luchesa et al. (16), the experimental group received TENS combined with conventional analgesia, whereas the study by Solak et al. (17) evaluated TENS combined with PCA. Solak et al. (17) reported two different pulse modulation modes of TENS (continuous and intermittent). Details are provided in Table 1. Finally, four datasets were included in the pooled results.

**Figure 5:**
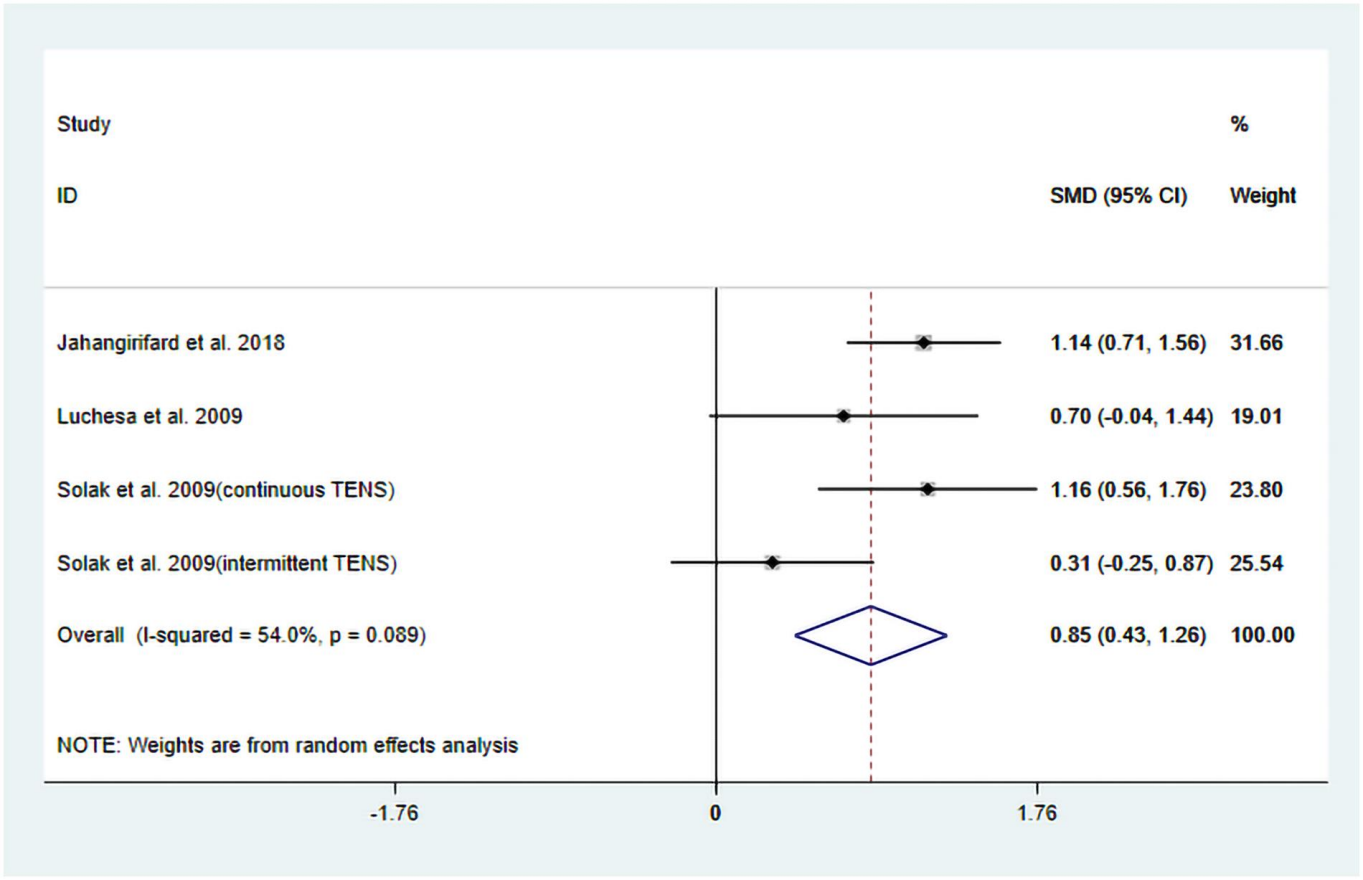
Resulting forest plot for FEV_1_.

The heterogeneity test results showed that *I*^2^ = 54.00% and P_H_ = 0.10. Given the limited number of included studies, the random-effects model was ultimately employed for the meta-analysis. The pooled analysis yielded the following: SMD = 0.85, 95% CI (0.43, 1.26), *Z* = 3.98, and *P* = 0.00. The results indicated that, compared to the control group, TENS significantly enhanced posttreatment FEV_1_ (Figure 5). Sensitivity analyses demonstrated that the findings of this study were robust (Supplementary Figure S6). However, publication bias results could not be assessed due to the limited number of included studies.

**Figure 6:**
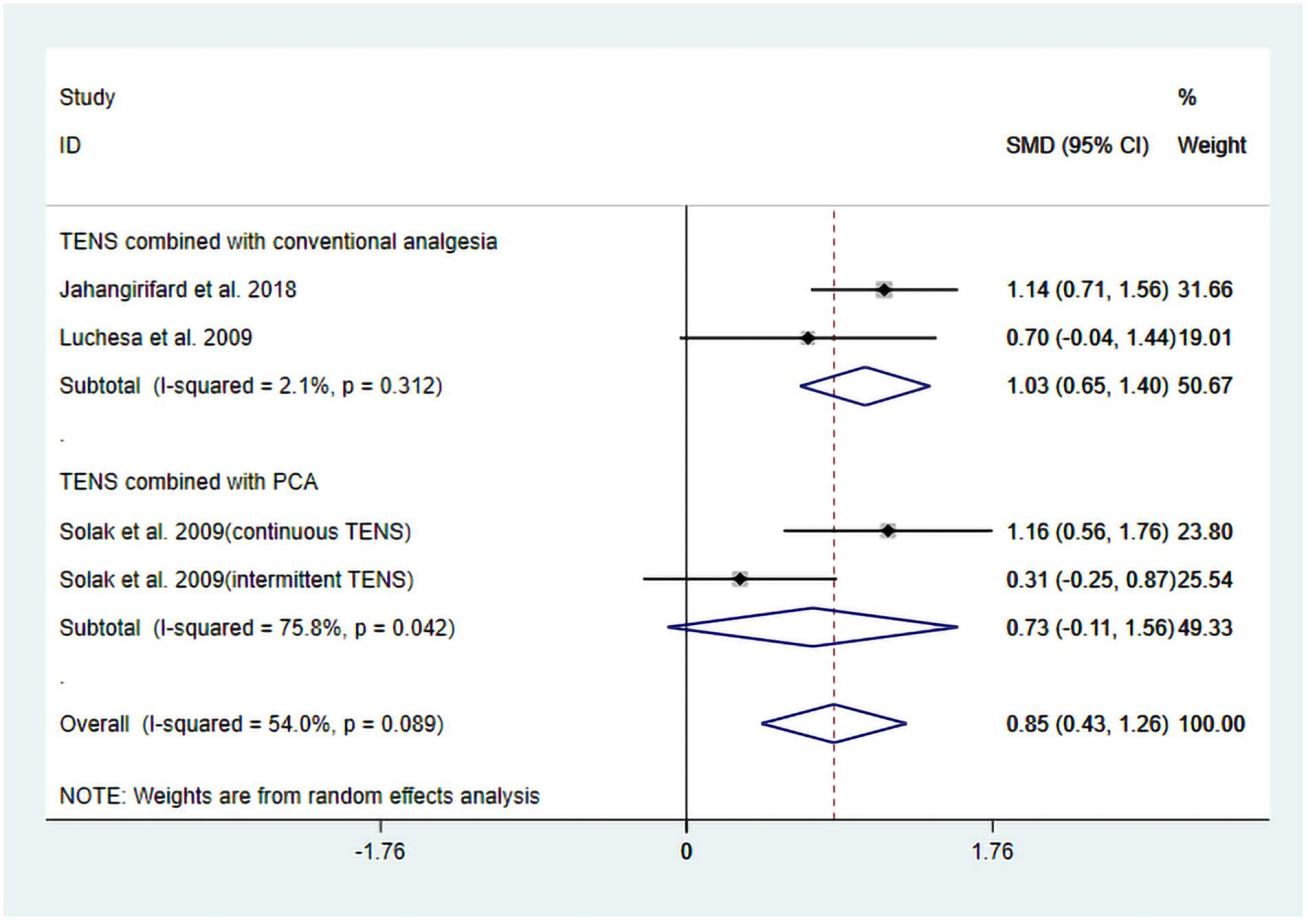
Forest plot of the results of subgroup analysis for FEV_1_.

Subgroup analyses were performed based on differences in intervention protocols within the experimental groups. The following results were obtained: when the intervention involved TENS combined with conventional analgesia, the pooled results demonstrated a significant improvement in FEV_1_ posttreatment: SMD = 1.03, 95% CI (0.65, 1.40); *Z* = 5.38, *P* = 0.00. In contrast, when the intervention involved a combination of TENS and PCA, the effect did not reach significant improvement in posttreatment FEV_1_: SMD = 0.72, 95% CI (−0.108, 1.56), *Z* = 1.71, *P* = 0.09 (Figure 6). Further meta-regression analysis indicated that the differences in intervention protocols within the experimental groups were not a significant source of heterogeneity.

##### 3.4.4.2 FVC

The studies by Jahangirifard et al. (15) and Luchesa et al. (16) evaluated FVC following TENS treatment. Two datasets were included in the meta-analysis.

**Figure 7:**
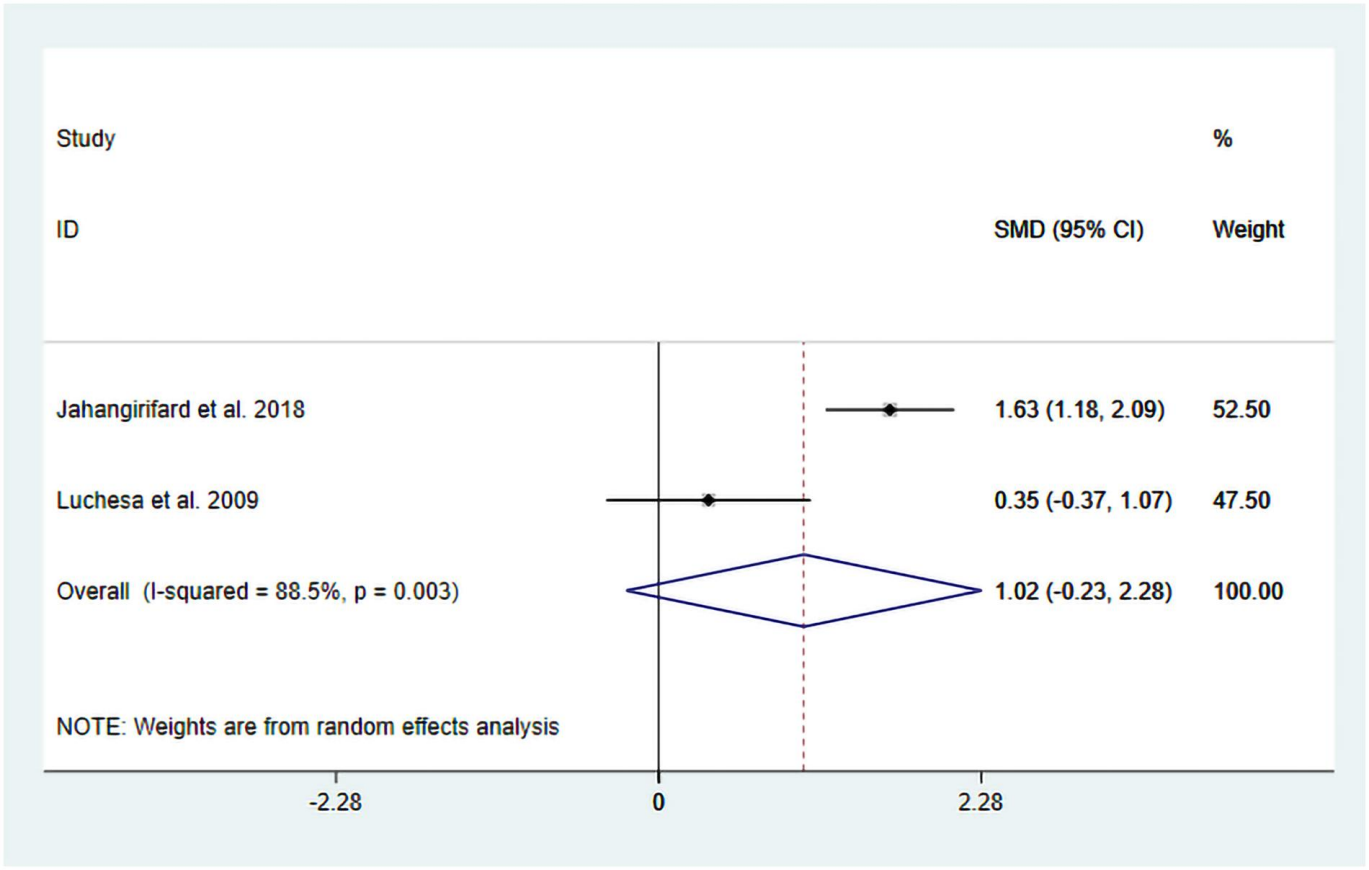
Resulting forest plot for FVC.

The heterogeneity test results showed that *I*^2^ = 88.50% and P_H_ = 0.003. Therefore, a random-effects model was used for pooling, and the pooled analysis yielded the following: SMD = −1.02, 95% CI (−0.23, 2.28), *Z* = 1.60, and *P* = 0.11. The results showed that, compared with the control group, TENS may reduce FVC, but there is no significant statistical significance (Figure 7). Sensitivity analyses indicated that the results of this study are robust (Supplementary Figure S7). Due to the insufficient number of included studies, the publication bias results cannot be assessed.

We observed a high degree of heterogeneity in this analysis. Although the intervention protocols were similar between the two studies, differences were present in the control group treatments. Specifically, Luchesa et al. (16) and others employed placebo TENS combined with conventional analgesic treatment, while another study used conventional analgesic treatment alone. Detailed information is given in Table 1. Therefore, these differences in control group intervention protocols may be one of the main reasons for the observed heterogeneity.

## 4 Discussion

This systematic review and meta-analysis evaluated the effects of TENS in patients undergoing CABG. Five RCTs were included, focusing on outcomes related to pain relief, anesthetic consumption, and pulmonary function. Although these results indicate that TENS can reduce anesthetic drug requirements after CABG surgery and improve FEV_1_, there is heterogeneity across the included studies, and some are limited by small sample sizes and short follow-up durations. These findings suggest that TENS can be used as a safe adjunctive therapy within multimodal rehabilitation programs after CABG surgery; however, more high-quality studies are needed to verify its long-term benefits and mechanism of action.

This systematic review and meta-analysis indicated that, compared to routine use of PCA alone following CABG surgery, the intervention of TENS could significantly decrease the overall consumption of anesthetic drugs postsurgery [SMD = −4.23, 95% CI (−7.31, −1.15), *Z* = 2.69, *P* = 0.007]. However, because the included studies used different types of opioids and none provided sufficient data to enable conversion to standardized morphine milligram equivalents (MME), we were unable to perform a meta-analysis based on absolute analgesic doses. Consequently, the SMD was used as the effect measure for statistical synthesis, which limits the clinical interpretability of this finding and indicates only a directional trend rather than a quantifiable clinical effect. Moreover, the heterogeneity of this result is quite high (*I^2^* = 96.20%), which cannot be easily ignored. In addition to the previously acknowledged issue of the lack of non-standardized dosing units, we also observed that differences in TENS stimulation protocols across studies—as well as variations in intervention duration, ranging from initiation at 24 h postoperatively to treatment courses lasting up to 5 days—may further contribute to the observed heterogeneity. Notably, the study by Solak et al. (17) conducted a time-based subgroup analysis and found that continuous TENS (CTENS) was more effectively than intermittent TENS (ITENS) in reducing postoperative morphine consumption, suggesting that the continuity of stimulation—i.e., differences in TENS protocols—is a key determinant of the opioid-sparing effect. Therefore, we consider the lack of standardized TENS protocols across studies to be a primary source of heterogeneity. Future trials should adopt MME as a core outcome measure and systematically investigate the impact of different TENS regimens on postoperative analgesic requirements following CABG to enhance the comparability and clinical applicability of findings.

However, regarding pain relief, both the VAS scores measured within 12 h and those assessed after 5 days indicated that TENS did not exhibit statistically significant differences. Although effect estimates consistently favored TENS (suggesting a potential trend toward pain relief), the results did not reach statistical significance (*p* > 0.05). This finding appears to contradict the aforementioned result that TENS significantly reduced postoperative analgesic consumption; however, when considered in the context of existing literature and methodological issues, more complex mechanisms underlie this discrepancy. For example, pain perception is influenced by multiple factors, such as psychological state, individual differences, and the type of surgery (18). Moreover, in some studies, TENS was used in combination with PCA, which may have produced a “ceiling effect” on pain relief and thereby obscured the independent contribution of TENS (19).

The results of this systematic review and meta-analysis also indicate that TENS has potential benefits in improving postoperative pulmonary function, particularly in achieving statistically significant results in increasing FEV_1_ (SMD = 0.85, *P* = 0.00). This finding holds potential clinical relevance. FEV_1_ is a key indicator for evaluating airway patency and lung function. Improvements in FEV_1_ may indicate that TENS is helpful in reducing postoperative airway obstruction, promoting mucus clearance, and thereby lowering the risk of pulmonary complications (such as pneumonia and atelectasis). Due to the limited number of studies included in this research, only subgroup analyses was feasible for the FEV_1_ outcome. It is noteworthy that subgroup analyses further elucidated the complexity of the mechanism: when TENS is combined with conventional analgesia, its effect on improving FEV_1_ is relatively pronounced (SMD = 1.03, *P* = 0.00). In contrast, when it is combined with PCA, the effect is not significant (SMD = 0.72, *P* = 0.09). Cardiac surgery is frequently associated with increase vagal nerve activity, leading to bronchial constriction and increased airway secretions, which in turn reduce FEV_1_ (20). TENS, especially high-frequency stimulation, is believed to activate Aβ fibers, which then affect the brainstem (such as the solitary nucleus and the vagal nucleus) through the ascending spinal pathway, thereby inhibiting the activity of the dorsal vagal nucleus and reducing cholinergic output to the airway smooth muscle, achieving bronchial dilation (21). Nevertheless, evidence suggests that postoperative pain and sedative medications can suppress the cough reflex, leading to retention of airway secretions and indirectly impairing FEV_1_ measurements (22). Therefore, in the context of conventional analgesia with low opioid exposure (e.g., Jahangirifard et al.), TENS may reduce the need for additional opioids, thereby preserving the cough reflex and diaphragmatic function, promoting airway secretion clearance, reducing the risk of atelectasis, and ultimately improving FEV_1_. In contrast, in PCA-based regimens (e.g., Solak et al.), even if TENS reduces the requirement for supplemental morphine, the persistently high opioid infusion may still lead to significant respiratory center depression and alterations in bronchial tone, thereby offsetting the potential airway benefits of TENS. Furthermore, this integrated mechanistic explanation indirectly provides theoretical support for our aforementioned findings that TENS intervention significantly reduces overall postoperative analgesic consumption but does not demonstrate a statistically significant difference in pain relief.

However, this study indicates that while TENS may reduce FVC (SMD = −1.02, *P* = 0.11), this result does not reach statistical significance. A possible reason is that FVC is an autonomous measurement that requires the patient to exert maximum effort and inherently includes confounding variables such as postoperative pain level, the sedative effects of analgesic drugs, and diaphragmatic fatigue. The time stratification in the study by Jahangirifard et al. (15) further complicated interpretation, with improvements in FVC ranging from MD = 3.00 to MD = 11.47 across different measurement time points. This variability suggests that TENS may have time-dependent effects on various components of respiratory function. Early postoperative benefits might be mediated by enhanced analgesia, which increases inspiratory effort, whereas later improvements could be attributable to reduced atelectasis due to improved cough efficiency. In contrast, the FEV_1_ component appears to be independent of the degree of effort—reflecting primarily large-airway dynamics rather than overall respiratory mechanics—and seems to be less sensitive to these confounding factors (23, 24). This characteristic might explain the lower heterogeneity for FEV₁ outcomes across the various trials (*I*^2^ = 54.00%). Therefore, the FEV_1_ analysis results may be considered more reliable than those of FVC.

Although this study independently analyzed the effects of TENS on pain relief, anesthetic drug consumption, and cardiac function recovery after CABG using meta-analytic methods, it seems to provide some evidence-based medical evidence to inform future research. However, the following limitations must be considered: First, the number of studies included in this research was relatively small, and significant heterogeneity was observed across results. Regarding the sources of this heterogeneity, although we conducted appropriate discussions and explorations in the Discussion section, we still could not provide a reasonable explanation from a statistical perspective. Second, the time measurement framework represents another key limitation, as the included studies indicated that time would affect the assessment of analgesic efficacy and pulmonary function. In addition, the short-term follow-up of the current trials (usually ≤7 days) precluded assessment of the long-term benefits. Third, the inability to standardize and quantify opioid use represents a major limitation of this study. The included studies used different opioids (e.g., morphine, pethidine) and did not provide sufficient data to enable conversion to MME. This substantially limits the clinical interpretability and translational value of the findings and may also contribute to the observed high heterogeneity. More refined studies are needed in the future to further explore ways to address these limitations and verify the outcomes of this study.

## 5 Conclusion

This systematic review and meta-analysis suggests that TENS may reduce postoperative analgesic consumption and significantly improve FEV₁ in patients undergoing CABG. However, the current evidence is limited by a small number of included studies, high methodological heterogeneity (e.g., variations in intervention parameters and inconsistent reporting of opioid types and dosages), short follow-up durations, and the lack of standardized MME data. Consequently, the definitive efficacy and optimal application implementation of TENS in post-CABG recovery remain uncertain. Future research should adopt standardized core outcome measures (such as MME) and consistent intervention protocols and conduct large-scale, long-term randomized controlled trials to clarify the clinical value of TENS.

## Data Availability

The datasets presented in this study can be found in online repositories. The names of the repository/repositories and accession number(s) can be found in the article/Supplementary Material.
