# Supplementary material for: Effect of transcutaneous electrical nerve stimulation on patients after coronary artery bypass grafting: a systematic review and meta-analysis
Source: Front Cardiovasc Med. 2026 Feb 5;13:1690565. doi: 10.3389/fcvm.2026.1690565 (PMC12916612; doi:10.3389/fcvm.2026.1690565)
Supplement: Supplementary file 1 [file Datasheet1.docx]

Supplementary Material

# Supplementary Data

## Details of the search

| **Supplementary Table 1.** Search Strategy | |
| --- | --- |
| **Pubmed** | |
| **Line #** | **Search** |
| 1 | **("Coronary Artery Bypass"[Mesh]) OR (((((((((((((Artery Bypass, Coronary) OR (Artery Bypasses, Coronary)) OR (Bypasses, Coronary Artery)) OR (Coronary Artery Bypasses)) OR (Coronary Artery Bypass Grafting)) OR (Coronary Artery Bypass Surgery)) OR (Aortocoronary Bypass)) OR (Aortocoronary Bypasses)) OR (Bypass, Aortocoronary)) OR (Bypasses, Aortocoronary)) OR (Bypass Surgery, Coronary Artery)) OR (Bypass, Coronary Artery)) OR (CABG))** |
| 2 | **("Transcutaneous Electric Nerve Stimulation"[Mesh]) OR (((((((((((((((((((((((((((((Transcutaneous Nerve Stimulation) OR (Nerve Stimulation, Transcutaneous)) OR (Stimulation, Transcutaneous Nerve)) OR (Electric Stimulation, Transcutaneous)) OR (Stimulation, Transcutaneous Electric)) OR (Transcutaneous Electric Stimulation)) OR (Percutaneous Electric Nerve Stimulation)) OR (TENS)) OR (Transdermal Electrostimulation)) OR (Electrostimulation, Transdermal)) OR (Percutaneous Electrical Nerve Stimulation)) OR (Transcutaneous Electrical Nerve Stimulation)) OR (Electrical Stimulation, Transcutaneous)) OR (Transcutaneous Electrical Stimulation)) OR (Analgesic Cutaneous Electrostimulation)) OR (Cutaneous Electrostimulation, Analgesic)) OR (Electrostimulation, Analgesic Cutaneous)) OR (Electroanalgesia)) OR (Electroanalgesias)) OR (Percutaneous Neuromodulation Therapy)) OR (Neuromodulation Therapy, Percutaneous)) OR (Percutaneous Neuromodulation Therapies)) OR (Therapy, Percutaneous Neuromodulation)) OR (Percutaneous Electrical Neuromodulation)) OR (Electrical Neuromodulation, Percutaneous)) OR (Electrical Neuromodulations, Percutaneous)) OR (Neuromodulation, Percutaneous Electrical)) OR (Neuromodulations, Percutaneous Electrical)) OR (Percutaneous Electrical Neuromodulations))** |
| 3 | **(("Coronary Artery Bypass"[Mesh]) OR (((((((((((((Artery Bypass, Coronary) OR (Artery Bypasses, Coronary)) OR (Bypasses, Coronary Artery)) OR (Coronary Artery Bypasses)) OR (Coronary Artery Bypass Grafting)) OR (Coronary Artery Bypass Surgery)) OR (Aortocoronary Bypass)) OR (Aortocoronary Bypasses)) OR (Bypass, Aortocoronary)) OR (Bypasses, Aortocoronary)) OR (Bypass Surgery, Coronary Artery)) OR (Bypass, Coronary Artery)) OR (CABG))) AND (("Transcutaneous Electric Nerve Stimulation"[Mesh]) OR (((((((((((((((((((((((((((((Transcutaneous Nerve Stimulation) OR (Nerve Stimulation, Transcutaneous)) OR (Stimulation, Transcutaneous Nerve)) OR (Electric Stimulation, Transcutaneous)) OR (Stimulation, Transcutaneous Electric)) OR (Transcutaneous Electric Stimulation)) OR (Percutaneous Electric Nerve Stimulation)) OR (TENS)) OR (Transdermal Electrostimulation)) OR (Electrostimulation, Transdermal)) OR (Percutaneous Electrical Nerve Stimulation)) OR (Transcutaneous Electrical Nerve Stimulation)) OR (Electrical Stimulation, Transcutaneous)) OR (Transcutaneous Electrical Stimulation)) OR (Analgesic Cutaneous Electrostimulation)) OR (Cutaneous Electrostimulation, Analgesic)) OR (Electrostimulation, Analgesic Cutaneous)) OR (Electroanalgesia)) OR (Electroanalgesias)) OR (Percutaneous Neuromodulation Therapy)) OR (Neuromodulation Therapy, Percutaneous)) OR (Percutaneous Neuromodulation Therapies)) OR (Therapy, Percutaneous Neuromodulation)) OR (Percutaneous Electrical Neuromodulation)) OR (Electrical Neuromodulation, Percutaneous)) OR (Electrical Neuromodulations, Percutaneous)) OR (Neuromodulation, Percutaneous Electrical)) OR (Neuromodulations, Percutaneous Electrical)) OR (Percutaneous Electrical Neuromodulations)))** |
| **Embase** | |
| **Line #** | **Search** |
| 1 | 'coronary artery bypass graft'/exp OR 'artery bypass, coronary':ab,ti OR 'artery bypasses, coronary':ab,ti OR 'bypasses, coronary artery':ab,ti OR 'coronary artery bypasses':ab,ti OR 'coronary artery bypass':ab,ti OR 'coronary artery bypass surgery':ab,ti OR 'aortocoronary bypass':ab,ti OR 'aortocoronary bypasses':ab,ti OR 'bypass, aortocoronary':ab,ti OR 'bypasses, aortocoronary':ab,ti OR 'bypass surgery, coronary artery':ab,ti OR 'bypass, coronary artery':ab,ti OR 'cabg':ab,ti |
| 2 | 'transcutaneous electrical nerve stimulation'/exp OR 'transcutaneous nerve stimulation':ab,ti OR 'nerve stimulation, transcutaneous':ab,ti OR 'stimulation, transcutaneous nerve':ab,ti OR 'electric stimulation, transcutaneous':ab,ti OR 'stimulation, transcutaneous electric':ab,ti OR 'transcutaneous electric stimulation':ab,ti OR 'percutaneous electric nerve stimulation':ab,ti OR 'tens':ab,ti OR 'transdermal electrostimulation':ab,ti OR 'electrostimulation, transdermal':ab,ti OR 'percutaneous electrical nerve stimulation':ab,ti OR 'transcutaneous electrical nerve stimulation':ab,ti OR 'electrical stimulation, transcutaneous':ab,ti OR 'transcutaneous electrical stimulation':ab,ti OR 'analgesic cutaneous electrostimulation':ab,ti OR 'cutaneous electrostimulation, analgesic':ab,ti OR 'electrostimulation, analgesic cutaneous':ab,ti OR 'electroanalgesia':ab,ti OR 'electroanalgesias':ab,ti OR 'percutaneous neuromodulation therapy':ab,ti OR 'neuromodulation therapy, percutaneous':ab,ti OR 'percutaneous neuromodulation therapies':ab,ti OR 'therapy, percutaneous neuromodulation':ab,ti OR 'percutaneous electrical neuromodulation':ab,ti OR 'electrical neuromodulation, percutaneous':ab,ti OR 'electrical neuromodulations, percutaneous':ab,ti OR 'neuromodulation, percutaneous electrical':ab,ti OR 'neuromodulations, percutaneous electrical':ab,ti OR 'percutaneous electrical neuromodulations':ab,ti |
| 3 | #1 AND #2 |
| **Cochrane Library** | |
| **Line #** | **Search** |
| 1 | MeSH descriptor: [Coronary Artery Bypass] explode all trees |
| 2 | ('Coronary Artery Bypass' OR 'Artery Bypass, Coronary' OR 'Artery Bypasses, Coronary' OR 'Bypasses, Coronary Artery' OR 'Coronary Artery Bypasses' OR 'Coronary Artery Bypass Grafting' OR 'Coronary Artery Bypass Surgery' OR 'Aortocoronary Bypass' OR 'Aortocoronary Bypasses' OR 'Bypass, Aortocoronary' OR 'Bypasses, Aortocoronary' OR 'Bypass Surgery, Coronary Artery' OR 'Bypass, Coronary Artery' OR 'CABG'):ti,ab,kw |
| 3 | #1 OR #2 |
| 4 | ('Transcutaneous Electric Nerve Stimulation' OR 'Transcutaneous Nerve Stimulation' OR 'Nerve Stimulation, Transcutaneous' OR 'Stimulation, Transcutaneous Nerve' OR 'Electric Stimulation, Transcutaneous' OR 'Stimulation, Transcutaneous Electric' OR 'Transcutaneous Electric Stimulation' OR 'Percutaneous Electric Nerve Stimulation' OR 'TENS' OR 'Transdermal Electrostimulation' OR 'Electrostimulation, Transdermal' OR 'Percutaneous Electrical Nerve Stimulation' OR 'Transcutaneous Electrical Nerve Stimulation' OR 'Electrical Stimulation, Transcutaneous' OR 'Transcutaneous Electrical Stimulation' OR 'Analgesic Cutaneous Electrostimulation' OR 'Cutaneous Electrostimulation, Analgesic' OR 'Electrostimulation, Analgesic Cutaneous' OR 'Electroanalgesia' OR 'Electroanalgesias' OR 'Percutaneous Neuromodulation Therapy' OR 'Neuromodulation Therapy, Percutaneous' OR 'Percutaneous Neuromodulation Therapies' OR 'Therapy, Percutaneous Neuromodulation' OR 'Percutaneous Electrical Neuromodulation' OR 'Electrical Neuromodulation, Percutaneous' OR 'Electrical Neuromodulations, Percutaneous' OR 'Neuromodulation, Percutaneous Electrical' OR 'Neuromodulations, Percutaneous Electrical' OR 'Percutaneous Electrical Neuromodulations'):ti,ab,kw |
| 5 | MeSH descriptor: [Transcutaneous Electric Nerve Stimulation] explode all trees |
| 6 | #4 OR #5 |
| 7 | #3 AND #6 |
| **Web of Science** | |
| **Line #** | **Search** |
| 1 | TS=(Coronary Artery Bypass OR Artery Bypass, Coronary OR Artery Bypasses, Coronary OR Bypasses, Coronary Artery OR Coronary Artery Bypasses OR Coronary Artery Bypass Grafting OR Coronary Artery Bypass Surgery OR Aortocoronary Bypass OR Aortocoronary Bypasses OR Bypass, Aortocoronary OR Bypasses, Aortocoronary OR Bypass Surgery, Coronary Artery OR Bypass, Coronary Artery OR CABG) |
| 2 | TS=(Transcutaneous Electric Nerve Stimulation OR Transcutaneous Nerve Stimulation OR Nerve Stimulation, Transcutaneous OR Stimulation, Transcutaneous Nerve OR Electric Stimulation, Transcutaneous OR Stimulation, Transcutaneous Electric OR Transcutaneous Electric Stimulation OR Percutaneous Electric Nerve Stimulation OR TENS OR Transdermal Electrostimulation OR Electrostimulation, Transdermal OR Percutaneous Electrical Nerve Stimulation OR Transcutaneous Electrical Nerve Stimulation OR Electrical Stimulation, Transcutaneous OR Transcutaneous Electrical Stimulation OR Analgesic Cutaneous Electrostimulation OR Cutaneous Electrostimulation, Analgesic OR Electrostimulation, Analgesic Cutaneous OR Electroanalgesia OR Electroanalgesias OR Percutaneous Neuromodulation Therapy OR Neuromodulation Therapy, Percutaneous OR Percutaneous Neuromodulation Therapies OR Therapy, Percutaneous Neuromodulation OR Percutaneous Electrical Neuromodulation OR Electrical Neuromodulation, Percutaneous OR Electrical Neuromodulations, Percutaneous OR Neuromodulation, Percutaneous Electrical OR Neuromodulations, Percutaneous Electrical OR Percutaneous Electrical Neuromodulations) |
| 3 | #1 AND #2 |

# Supplementary Figures and Tables

## Supplementary Figures


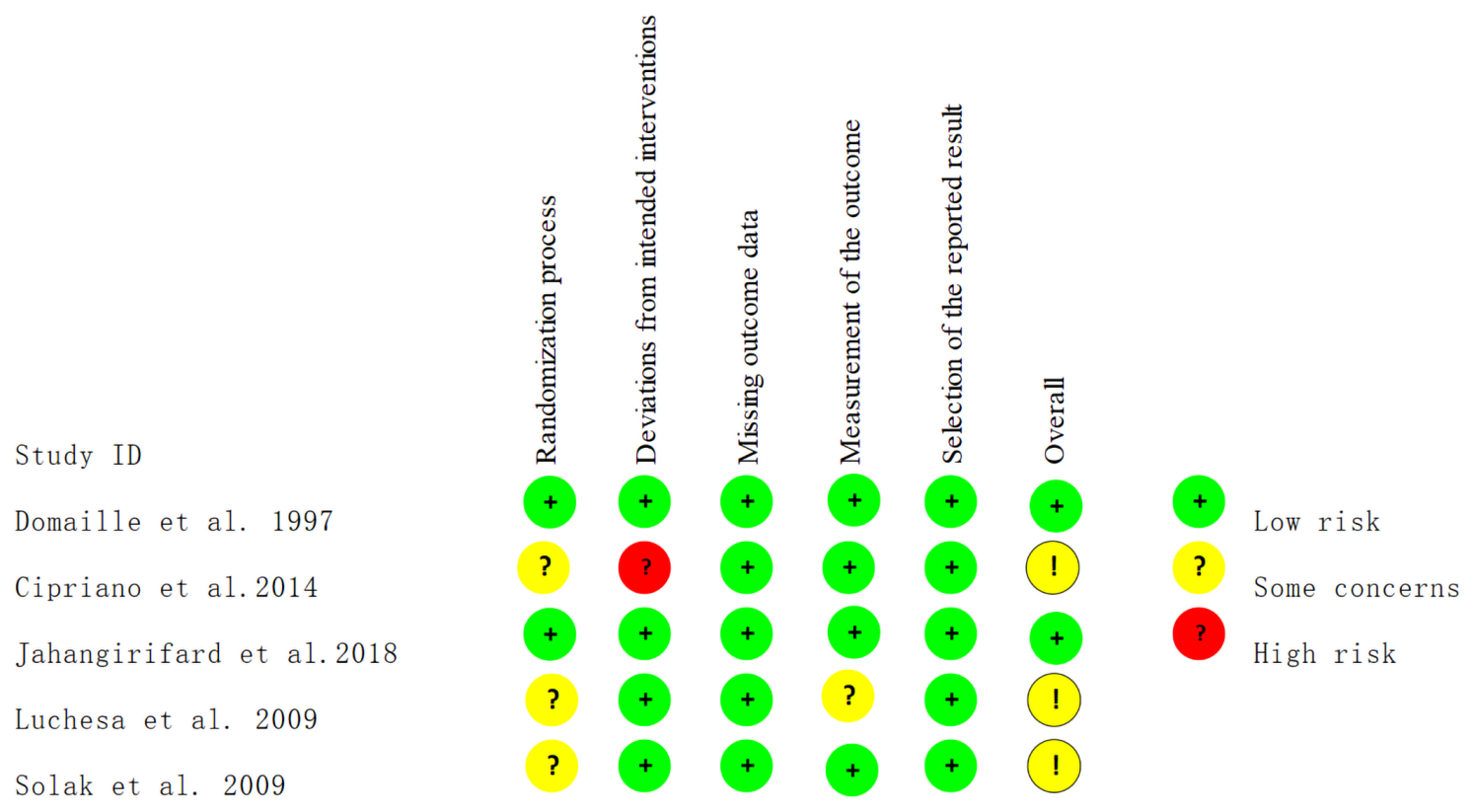


**Supplementary Figures 1.** Detailed results of RCT quality assessment


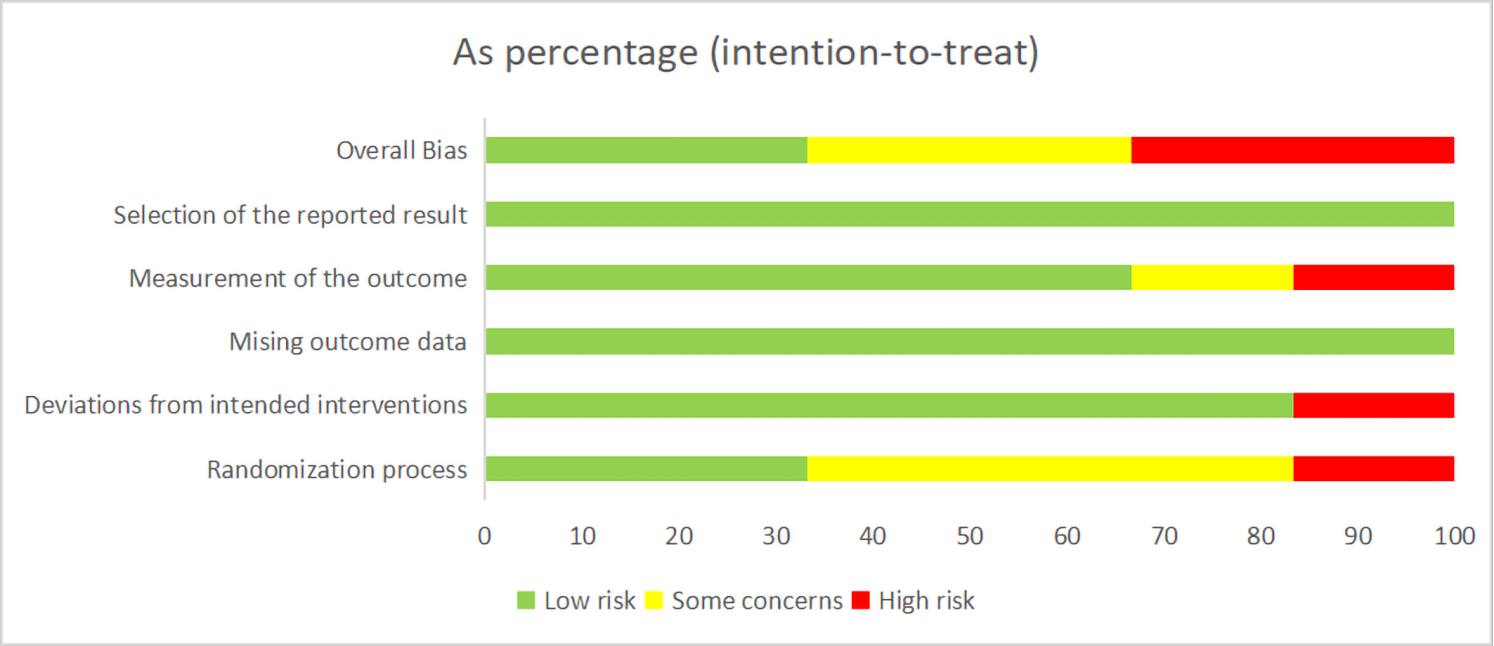


**Supplementary Figures 2.** Proportion chart of RCT quality assessment


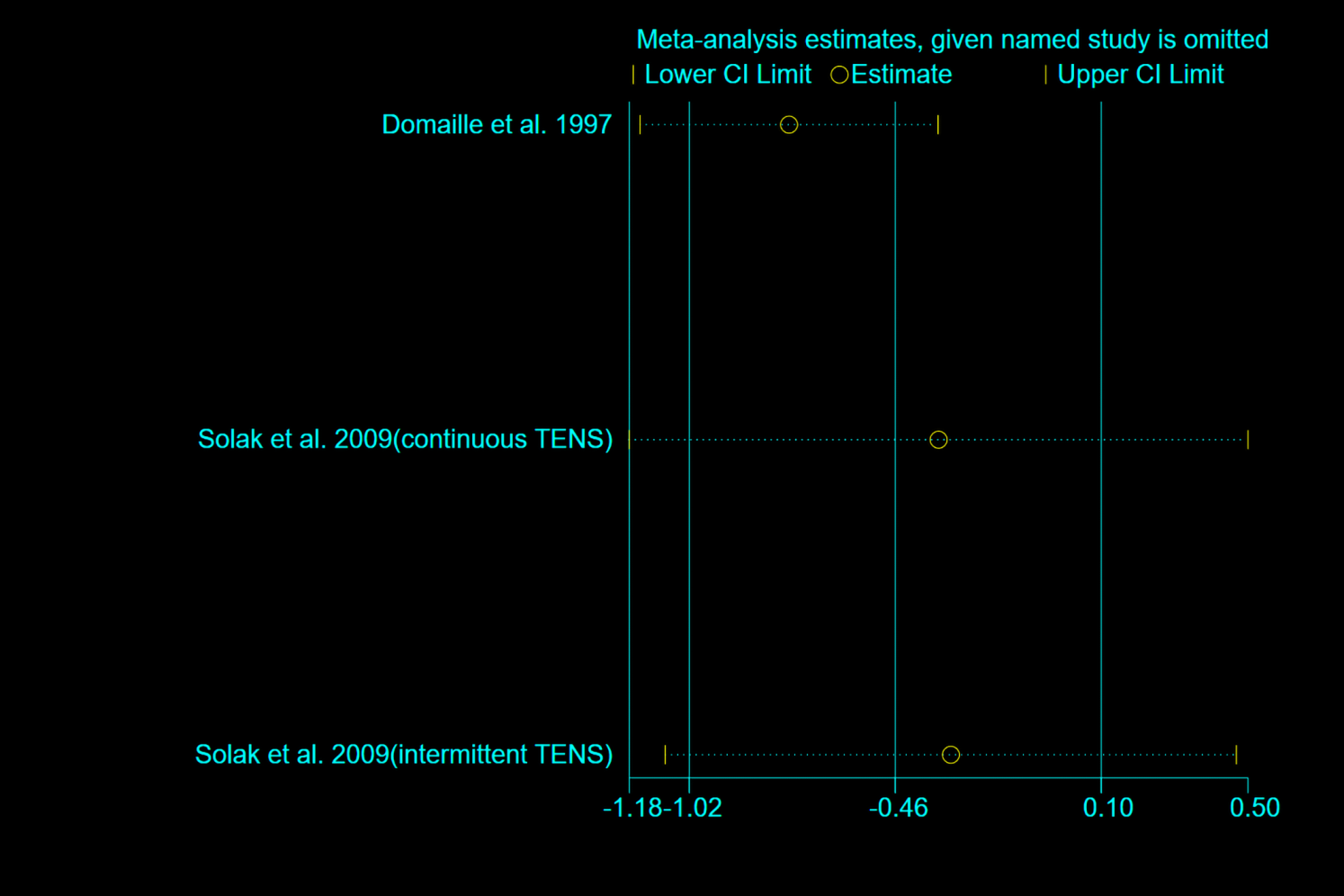


**Supplementary Figure 3.** Results of Sensitivity Analyses on Postoperative Acute Pain at Rest (within 12 Hours)


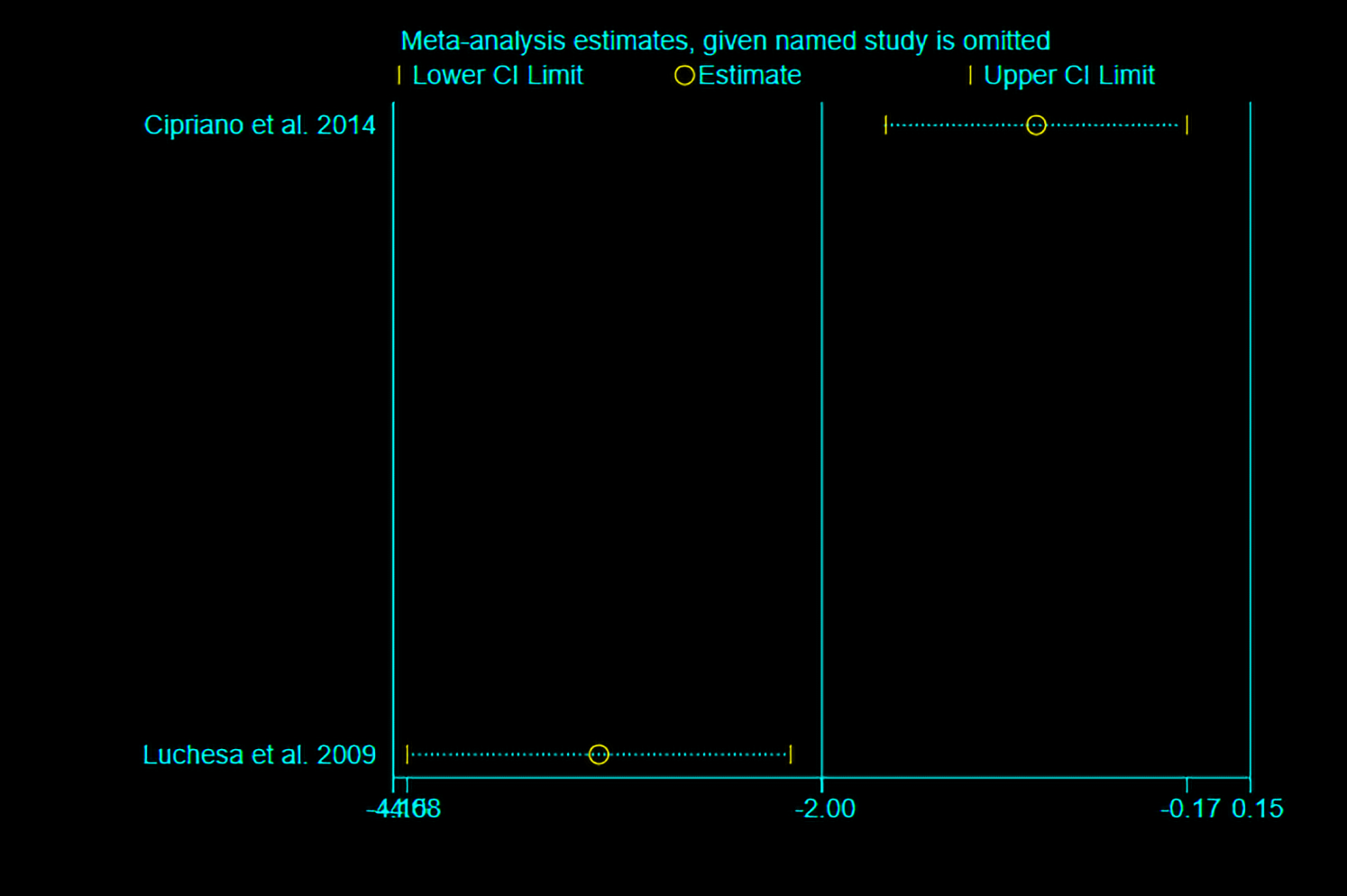


**Supplementary Figure 4.** Results of Sensitivity Analyses on Postoperative Chronic Pain at Rest (5 Days)


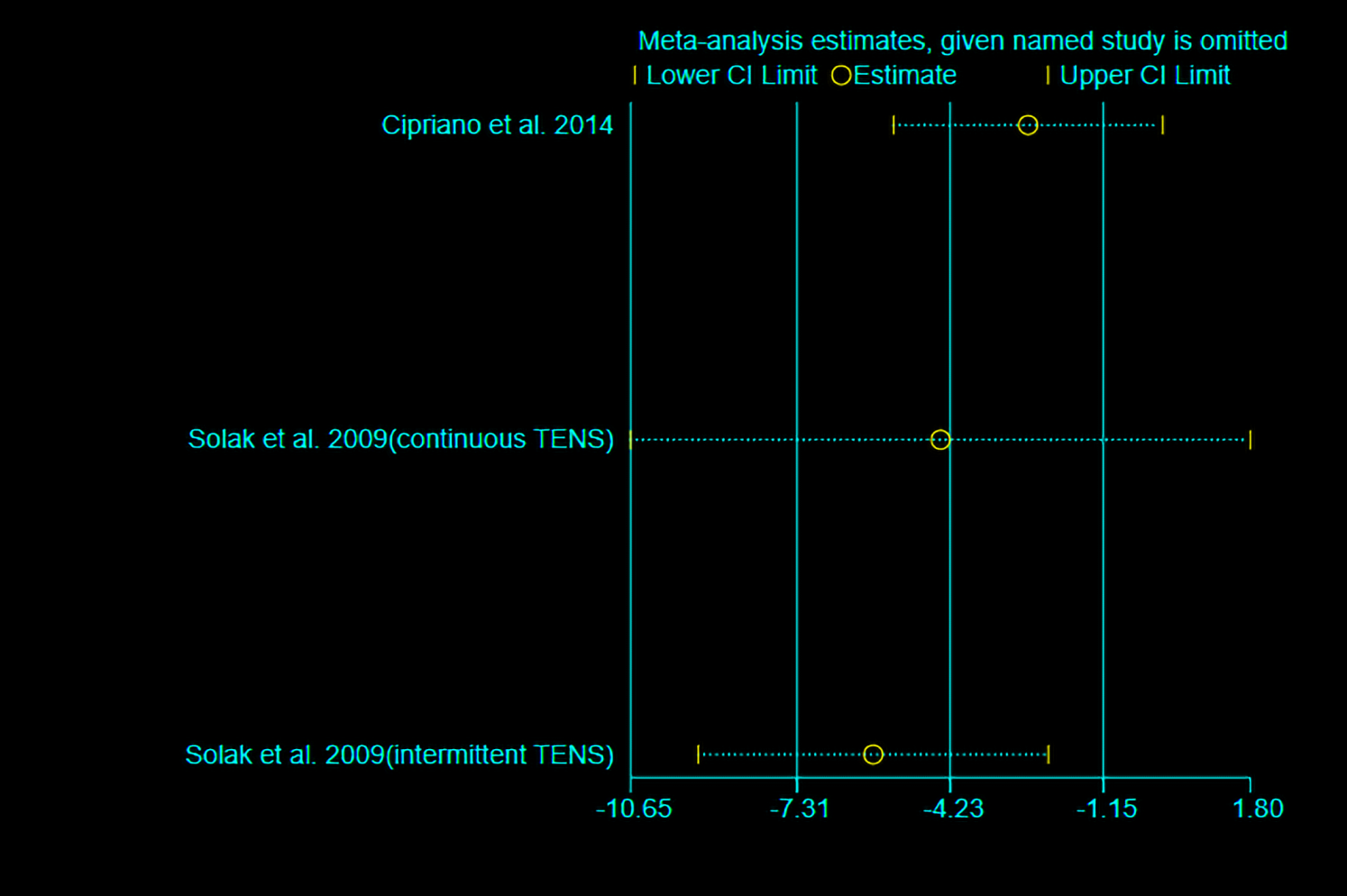


**Supplementary Figure 5.** Results of Sensitivity Analyses on Postoperative Pain Medication Consumption


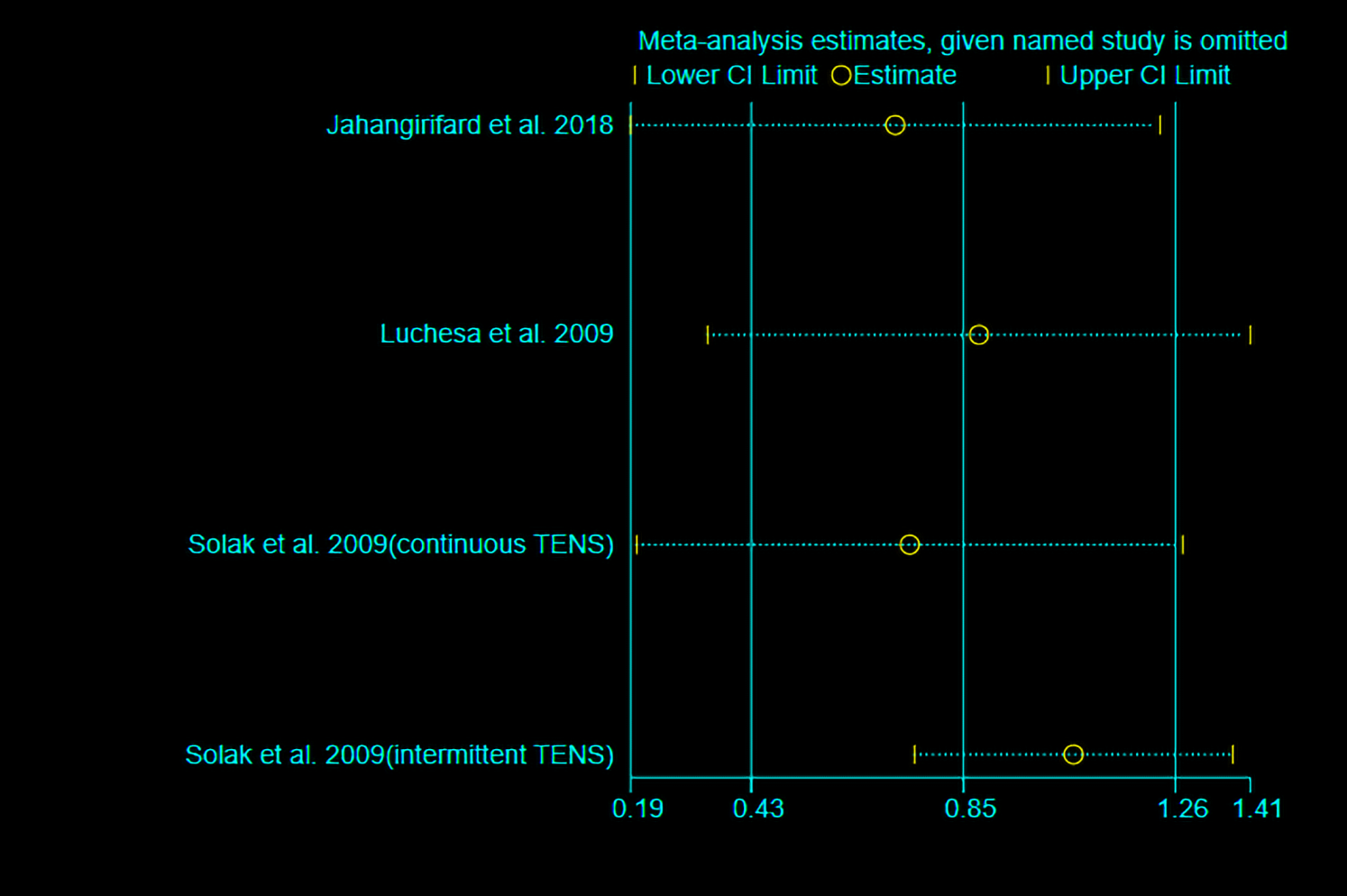


**Supplementary Figure 6.** Results of Sensitivity Analyses on FEV_1_


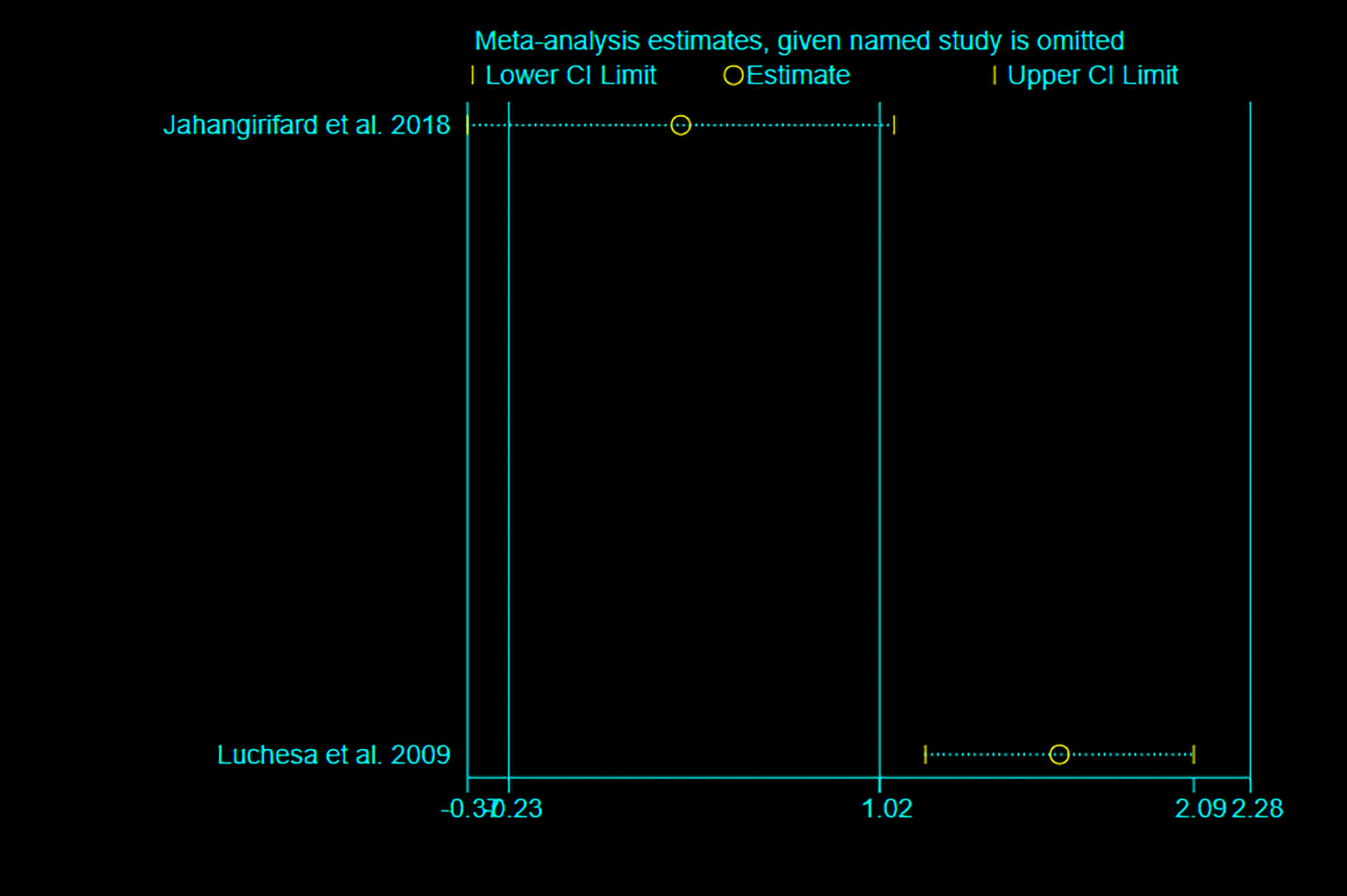


**Supplementary Figure 7.** Results of Sensitivity Analyses on FVC

## Supplementary Tables

**Supplementary Table 2.** GRADE evaluation results of evidence levels

| **Quality assessment** | | | | | | | **No of patients** | | **Effect** | | **Quality** | **Importance** |
| --- | --- | --- | --- | --- | --- | --- | --- | --- | --- | --- | --- | --- |
|  |  |  |  |  |  |  |  |  |  |  |  |  |
| **No of studies** | **Design** | **Risk of bias** | **Inconsistency** | **Indirectness** | **Imprecision** | **Other considerations** | **TENS** | **Sham Electrical Stimulation** | **Relative (95% CI)** | **Absolute** |  |  |
| **Postoperative acute pain at rest (within 12 hours) (measured with: Postoperative acute pain at rest (within 12 hours); Better indicated by lower values)** | | | | | | | | | | | | |
| 2 | randomised trials | no serious risk of bias | no serious inconsistency^1^ | no serious indirectness | serious^1^ | none^1^ | 56 | 53 | - | SMD 0.46 lower (1.02 lower to 0.1 higher) | ÅÅÅO MODERATE | CRITICAL |
| **Postoperative chronic (5 days) pain at rest (measured with: Postoperative chronic (5 days) pain at rest ; Better indicated by lower values)** | | | | | | | | | | | | |
| 2 | randomised trials | no serious risk of bias^1^ | no serious inconsistency | no serious indirectness^1^ | serious^1^ | none | 35 | 32 | - | SMD 2.00 lower (4.15 lower to 0.146 higher) | ÅÅÅO MODERATE | CRITICAL |
| **FEV1 (measured with: FEV1; Better indicated by lower values)** | | | | | | | | | | | | |
| 3 | randomised trials | no serious risk of bias^1^ | no serious inconsistency | no serious indirectness | serious^1^ | none | 115 | 115 | - | SMD 0.85 higher (0.43 to 1.26 higher) | ÅÅÅO MODERATE | IMPORTANT |
| **FVC (measured with: FVC; Better indicated by lower values)** | | | | | | | | | | | | |
| 2 | randomised trials | no serious risk of bias^1^ | no serious inconsistency | no serious indirectness | serious^1^ | none | 65 | 65 | - | SMD 1.02 lower (0.23 lower to 2.28 higher) | ÅÅÅO MODERATE | IMPORTANT |
| **Postoperative pain medication consumption (measured with: Postoperative pain medication consumption; Better indicated by lower values)** | | | | | | | | | | | | |
| 2 | randomised trials | no serious risk of bias^1^ | no serious inconsistency | no serious indirectness | serious^1^ | none | 70 | 68 | - | SMD 4.23 lower (7.31 to 1.15 lower) | ÅÅÅO MODERATE | CRITICAL |

^1^ No explanation was provided

**Supplementary Table 3.** PRISMA_2020_abstract_checklist

| **Section and Topic** | **Item #** | **Checklist item** | **Location where item is reported** |
| --- | --- | --- | --- |
| **TITLE** | | |  |
| Title | 1 | Identify the report as a systematic review. | Title |
| **ABSTRACT** | | |  |
| Abstract | 2 | See the PRISMA 2020 for Abstracts checklist. | Abstract |
| **INTRODUCTION** | | |  |
| Rationale | 3 | Describe the rationale for the review in the context of existing knowledge. | Introduction |
| Objectives | 4 | Provide an explicit statement of the objective(s) or question(s) the review addresses. | Introduction |
| **METHODS** | | |  |
| Eligibility criteria | 5 | Specify the inclusion and exclusion criteria for the review and how studies were grouped for the syntheses. | Materials and Methods |
| Information sources | 6 | Specify all databases, registers, websites, organisations, reference lists and other sources searched or consulted to identify studies. Specify the date when each source was last searched or consulted. | Materials and Methods |
| Search strategy | 7 | Present the full search strategies for all databases, registers and websites, including any filters and limits used. | Materials and Methods |
| Selection process | 8 | Specify the methods used to decide whether a study met the inclusion criteria of the review, including how many reviewers screened each record and each report retrieved, whether they worked independently, and if applicable, details of automation tools used in the process. | Materials and Methods |
| Data collection process | 9 | Specify the methods used to collect data from reports, including how many reviewers collected data from each report, whether they worked independently, any processes for obtaining or confirming data from study investigators, and if applicable, details of automation tools used in the process. | Materials and Methods |
| Data items | 10a | List and define all outcomes for which data were sought. Specify whether all results that were compatible with each outcome domain in each study were sought (e.g. for all measures, time points, analyses), and if not, the methods used to decide which results to collect. | Materials and Methods |
|  | 10b | List and define all other variables for which data were sought (e.g. participant and intervention characteristics, funding sources). Describe any assumptions made about any missing or unclear information. | Materials and Methods |
| Study risk of bias assessment | 11 | Specify the methods used to assess risk of bias in the included studies, including details of the tool(s) used, how many reviewers assessed each study and whether they worked independently, and if applicable, details of automation tools used in the process. | Materials and Methods |
| Effect measures | 12 | Specify for each outcome the effect measure(s) (e.g. risk ratio, mean difference) used in the synthesis or presentation of results. | Materials and Methods |
| Synthesis methods | 13a | Describe the processes used to decide which studies were eligible for each synthesis (e.g. tabulating the study intervention characteristics and comparing against the planned groups for each synthesis (item #5)). | Materials and Methods |
|  | 13b | Describe any methods required to prepare the data for presentation or synthesis, such as handling of missing summary statistics, or data conversions. | Materials and Methods |
|  | 13c | Describe any methods used to tabulate or visually display results of individual studies and syntheses. | Materials and Methods |
|  | 13d | Describe any methods used to synthesize results and provide a rationale for the choice(s). If meta-analysis was performed, describe the model(s), method(s) to identify the presence and extent of statistical heterogeneity, and software package(s) used. | Materials and Methods |
|  | 13e | Describe any methods used to explore possible causes of heterogeneity among study results (e.g. subgroup analysis, meta-regression). | Materials and Methods |
|  | 13f | Describe any sensitivity analyses conducted to assess robustness of the synthesized results. | Materials and Methods |
| Reporting bias assessment | 14 | Describe any methods used to assess risk of bias due to missing results in a synthesis (arising from reporting biases). | Materials and Methods |
| Certainty assessment | 15 | Describe any methods used to assess certainty (or confidence) in the body of evidence for an outcome. | Materials and Methods |
| **RESULTS** | | |  |
| Study selection | 16a | Describe the results of the search and selection process, from the number of records identified in the search to the number of studies included in the review, ideally using a flow diagram. | Results |
|  | 16b | Cite studies that might appear to meet the inclusion criteria, but which were excluded, and explain why they were excluded. | Results |
| Study characteristics | 17 | Cite each included study and present its characteristics. | Results |
| Risk of bias in studies | 18 | Present assessments of risk of bias for each included study. | Results |
| Results of individual studies | 19 | For all outcomes, present, for each study: (a) summary statistics for each group (where appropriate) and (b) an effect estimate and its precision (e.g. confidence/credible interval), ideally using structured tables or plots. | Results |
| Results of syntheses | 20a | For each synthesis, briefly summarise the characteristics and risk of bias among contributing studies. | Results |
|  | 20b | Present results of all statistical syntheses conducted. If meta-analysis was done, present for each the summary estimate and its precision (e.g. confidence/credible interval) and measures of statistical heterogeneity. If comparing groups, describe the direction of the effect. | Results |
|  | 20c | Present results of all investigations of possible causes of heterogeneity among study results. | Results |
|  | 20d | Present results of all sensitivity analyses conducted to assess the robustness of the synthesized results. | Results |
| Reporting biases | 21 | Present assessments of risk of bias due to missing results (arising from reporting biases) for each synthesis assessed. | Results |
| Certainty of evidence | 22 | Present assessments of certainty (or confidence) in the body of evidence for each outcome assessed. | Results |
| **DISCUSSION** | | |  |
| Discussion | 23a | Provide a general interpretation of the results in the context of other evidence. | Discussion |
|  | 23b | Discuss any limitations of the evidence included in the review. | Discussion |
|  | 23c | Discuss any limitations of the review processes used. | Discussion |
|  | 23d | Discuss implications of the results for practice, policy, and future research. | Discussion |
| **OTHER INFORMATION** | | |  |
| Registration and protocol | 24a | Provide registration information for the review, including register name and registration number, or state that the review was not registered. | Materials and Methods |
|  | 24b | Indicate where the review protocol can be accessed, or state that a protocol was not prepared. | Materials and Methods |
|  | 24c | Describe and explain any amendments to information provided at registration or in the protocol. | Materials and Methods |
| Support | 25 | Describe sources of financial or non-financial support for the review, and the role of the funders or sponsors in the review. | Funding |
| Competing interests | 26 | Declare any competing interests of review authors. | Conflict of Interest |
| Availability of data, code and other materials | 27 | Report which of the following are publicly available and where they can be found: template data collection forms; data extracted from included studies; data used for all analyses; analytic code; any other materials used in the review. | Supplementary Material |
